# Continuous-wave laser absorption spectroscopy of the thorium-229 nucleus

**DOI:** 10.1038/s41586-026-11011-7

**Published:** 2026-09-16

**Authors:** I. Morawetz, T. Riebner, L. Toscani De Col, F. Schneider, N. Sempelmann, F. Schaden, M. Bartokos, G. A. Kazakov, S. Lahs, K. Beeks, B. Gerstenecker, A. Grüneis, M. Pimon, T. Schumm, V. Lal, G. Zitzer, V. Petrov, J. Tiedau, M. V. Okhapkin, E. Peik

**Affiliations:** 1 Vienna Center for Quantum Science and Technology, Atominstitut, TU Wien, Vienna, Austria; 2 Bundesamt für Eich- und Vermessungswesen (BEV), Vienna, Austria; 3 Physikalisch-Technische Bundesanstalt (PTB), Braunschweig, Germany; 4 Max-Born-Institute for Nonlinear Optics and Ultrafast Spectroscopy, Berlin, Germany

**Keywords:** Quantum metrology, Solid-state lasers, Experimental nuclear physics, Fluorescence spectroscopy

## Abstract

The low-energy nuclear transition in thorium-229 (Th-229) has been excited in thorium-doped crystals with laser light^1–3^, opening the path towards a highly stable and robust solid-state optical nuclear clock^4^. The required laser radiation at 148-nm wavelength has so far been produced using pulsed laser systems, in which only a small fraction of the incident photons has been resonant with the narrow nuclear transition. Here we show that the nucleus can be excited with a continuous-wave (CW), narrow-bandwidth, solid-state laser source^5^ at sub-nanowatt power and that the nuclear resonance signal can be detected in absorption rather than fluorescence. This eliminates the slow nuclear fluorescence decay from the detection process, allowing for clock operation with fast signal acquisition. We characterize two different thorium centres in a calcium fluoride (CaF_2_) crystal and measure the isomeric shift between them. One of the centres shows a very small static electric crystal field gradient <0.1 V Å^−2^ compared with gradients in the range of 100 V Å^−2^ observed previously^3,6^. This indicates a centre with high symmetry of the ions surrounding the thorium nucleus, promising nuclear resonance lines that are nearly independent of the lattice spacing.

## Abstract (web summary)

Absorption spectroscopy of thorium-229 nuclear excited states in a thorium-doped calcium fluoride crystal demonstrates a path towards a much more efficient way to build a highly stable and robust solid-state optical nuclear clock.

## Main

The 8.4-eV low-energy nuclear transition of Th-229 is investigated for the application as an optical nuclear clock of very high accuracy and stability^1–4,6–8^. The nuclear resonance may be examined with thorium ions in an ion trap in vacuum or inside a transparent crystal. In solids, Th-229 becomes a test case for laser Mössbauer spectroscopy that is sensitive to the interaction between the nucleus and its environment^3,6^. In the first experiments on laser excitation of Th-229, the required coherent vacuum ultraviolet (VUV) radiation at 148-nm wavelength was generated using four-wave mixing (FWM)^1,2,6,9^ or high-harmonic generation (HHG)^3^ of pulsed laser sources. In these experiments, only a small fraction of the VUV photons were in resonance with the thorium nuclei.

For the sources based on FWM, the spectral width was several orders of magnitude wider than the nuclear linewidth in the host crystals used^1,2,6^. For the HHG source using a femtosecond frequency comb, the crystal-field-broadened nuclear linewidth in the range 10–100 kHz was well resolved^10^, but on the order of 10^5^ non-resonant comb modes were present, whereas only a single mode contributed to the signal^3,10^. Laser excitation was detected by observing the nuclear fluorescence, slowly decaying with a time constant of about 600 s after blocking the radiation impinging on the crystal. Exploiting the full potential of a Th-229 solid-state nuclear clock requires a laser source with a linewidth comparable with the crystal-field-broadened linewidth and a fast, sensitive and robust detection method. These requirements can be fulfilled with the use of a narrow-linewidth CW laser, which—in contrast to HHG—combines the full laser power within the nuclear transition and therefore enables direct laser absorption spectroscopy with much higher signal-to-noise ratio, as demonstrated in this work.

Coherent VUV light can be generated through frequency conversion of laser radiation starting at longer wavelengths. The task is complex because only a few nonlinear optical materials are transparent in the VUV spectral region and have the properties for phase-matched frequency conversion. Recently, two different CW laser sources at the nuclear resonance wavelength of Th-229 have been reported: an all-solid-state system based on three consecutive steps of second-harmonic generation (SHG)^5^ starting from an infrared diode laser at 1,187 nm and a system based on FWM in cadmium vapour^11^. Although the FWM system produced about 100 times higher VUV power, the solid-state system is more compact and requires the frequency stabilization of only one single laser at a technically convenient wavelength of 1,187 nm that can be easily linked with other optical clocks. An essential element of the CW system is the use of a strontium tetraborate (SrB_4_O_7_, SBO) crystal for frequency doubling to 148 nm (refs. ^5,12^).

## Nuclear absorption method

A narrow-linewidth CW laser, with a linewidth comparable with or narrower than the nuclear transition in the host crystal, has the advantage that all photons can be resonant with the transition. Therefore, the nuclear excitation can be detected in absorption by measuring the attenuation of laser power transmitted through the crystal. A spectroscopic signal can be recorded by modulating the laser frequency over a fraction of the absorption linewidth and detecting the changes in transmitted power in phase correlation with the modulation frequency (see refs. ^13,14^ for example).

**Fig. 1:**
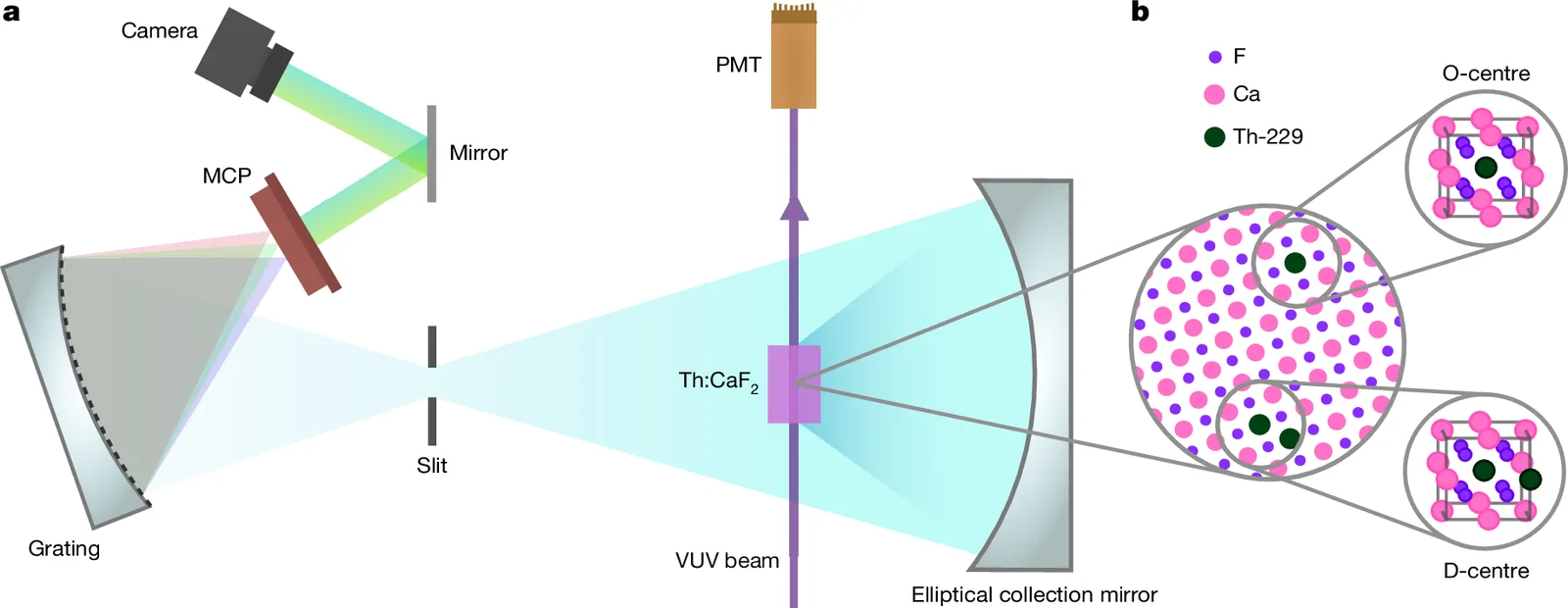
Illustration of the detection set-up and the microscopic structure of investigated Th-229 doping centres in the CaF_2_ host crystal. **a**, For the fluorescence detection, the signal is focused by a collection mirror, diffracted by a spherical concave grating and detected by a MCP detector with a phosphor screen. The phosphorescence is finally detected using a CMOS camera with an objective. For absorption measurements, a CsI PMT is positioned behind the Th:CaF_2_ crystal. **b**, Crystal structure of Th:CaF_2_ with the two investigated doping centres. The D-centre is a thorium dimer configuration with dihedral symmetry and the O-centre is a high-O_h_-symmetry configuration.

The ability to perform absorption detection is pivotal for the operation of a solid-state nuclear clock. The absorption detection examines the nuclear excitation, depleting a small fraction of the ground-state population, resulting in the attenuation of a directed laser beam while passing the sample. The measurement is effectively immediate, the timing of the interrogation sequences is determined by optimization of the signal-to-noise ratio, not constrained by the Th-229 excited-state lifetime of about 600 s in CaF_2_ host crystals^1,3^. The coherence time for the Th-229 resonance in fluoride crystals is estimated to be less than 10 ms, being greatly reduced owing to the magnetic dipole interaction of the Th-229 with the surrounding fluorine-19 nuclei^15,16^ (Fig. 1). This sets the maximum excitation time that will lead to the smallest spectroscopic linewidth.

The minimum time for fluorescence detection of the Th-229 nuclei is determined by the desired signal-to-noise ratio and by the time constant of the radiative decay. In preparation of the next interrogation cycle, a waiting time may be required to let the excited-state population return to the ground state, also determined by the radiative decay, potentially accelerated by laser-induced quenching into the range 100 s (refs. ^6,17,18^) or 10 s by X-ray-induced quenching^19,20^. This imposes an inefficient operation cycle for the clock in which most of the time would be spent for detection and state reinitialization and only a small fraction of time for interrogation, when the oscillator is actually compared with the nuclear reference and its frequency excursions can be corrected^16^. Absorption detection improves on the above as the temporal response of the signal is not limited by the long time constant of the radiative decay and the signal is detected simultaneously with the excitation. The use of a narrow-linewidth CW laser and absorption detection offers further benefits in comparison with the pulsed laser excitation and fluorescence detection used so far. It minimizes the detrimental effects on the signal from non-resonant VUV photons. These effects may comprise AC Stark shift, quenching of the population of the excited state^6,17,18^ and radiation damage of the crystal^21^. Any process in which resonant nuclear excitation is followed by non-radiative decay (such as shown in ref. ^8^) fully contributes to the absorption signal, while remaining undetected in fluorescence. The full spectroscopic information is contained in the amplitude and phase of the transmitted laser beam. Apertures can be used to shield the photodetector from the background of Cherenkov radiation and radioluminescence that is emitted isotropically from the thorium-doped crystal. No bulky VUV collection optics are required as for fluorescence detection, in which a large solid angle needs to be covered to obtain high sensitivity.

The enhanced sensitivity and rapid response of CW laser spectroscopy allows us to perform high-resolution laser Mössbauer studies of different thorium dopant sites in CaF_2_ and to compare their absorption and excitation spectra. This enables studies on site-specific frequency shift and broadening of nuclear transition lines, temperature dependencies and effects of external fields and material strain. Laser absorption spectroscopy provides a direct quantitative measurement of the nuclear column density of a specific doping centre; it also allows to detect centres that do not decay through VUV radiative decay (or on too short timescales).

## Fluorescence and absorption set-up

The experimental set-up consists of a VUV laser source and a nuclear spectroscopy vacuum chamber with thorium-doped calcium fluoride (Th:CaF_2_).

The Th:CaF_2_ crystal is a segment of the X2 sample^22^. Other pieces of the same ingot were previously used in refs. ^1,3,6,10,17,19,20,23,24^. The crystal has a cylindrical geometry with a 3.1(1)-mm diameter and 4.2(1)-mm length and is oriented such that the laser traverses along the line centre. The bulk doping concentration of Th-229, obtained by γ-spectroscopy and weighing, is determined to be 6.6(5) × 10^15^ mm^−3^. A spread of concentration within grown crystals of 10% was observed in the radial as well as axial directions; the local concentration examined by the laser may therefore vary more than the indicated uncertainty. During all experiments reported here, the crystal temperature was kept at 294.7(5) K. The optical transmission of the crystal in the range of several nanometres around the nuclear transition wavelength was measured to be 40(5)% using a VUV monochromator in a broad spectral range^21,22^.

A high-power laser at 1,187 nm is frequency-quadrupled to about 500 mW of 296.8-nm radiation. This radiation is used in the final single-pass SHG step from 296.8 nm to 148.4 nm. This step is based on SHG in a random quasi-phase-matched^12,25^ SBO crystal placed in a vacuum chamber. For stable SHG output power, the SBO crystal is kept under a high-purity N_2_ (purity 5.0) environment at 1,013-mbar pressure. The chamber with the SBO crystal is separated from the vacuum beamline by a MgF_2_ viewport. The VUV beam is guided to the spectroscopy chamber and aligned to the Th-229 crystal using a photomultiplier tube (PMT) positioned behind the crystal (Fig. 1). A movable mirror allows to redirect the beams into a VUV spectrometer (HP Spectroscopy easyLIGHT) for diagnostics and power measurements. About 1 nW of VUV power at 148.4 nm is generated from 350 mW of fundamental power^5^. It is reduced owing to losses from three dichroic mirrors, two MgF_2_ viewports and a collimation lens. Further losses appear owing to Fresnel reflections, substantial scattering from both crystal facets and the VUV transmission of the Th:CaF_2_ crystal. Therefore, the final VUV optical power measured after the crystal is approximately 70 pW.

Initially, an overview VUV spectrum of the nuclear quadrupole structure is recorded using the TA-FHG pro laser, frequency stabilized at the wavelength of 1,187 nm to a relevant mode of an infrared frequency comb (Menlo FC1500-250-ULN) by an offset-frequency phase lock. The repetition rate of the frequency comb is stabilized by locking another comb mode to a high-finesse cavity-stabilized external-cavity laser (Menlo ORS) at 1,542 nm located at the TU Wien Atominstitut (ATI). The VUV radiation linewidth in this operation mode is on the order of 300 kHz (see Methods for explanations). The frequency lock of the TA-FHG laser to a comb mode is used for an initial search and a wide scan of the fluorescence and absorption spectra.

Further, for better spectral resolution of quadrupole lines required for the operation of a solid-state nuclear clock, the TA-FHG pro laser is phase locked to the radiation of a high-finesse cavity-stabilized external-cavity diode laser (ECDL) at 1,187 nm, which has an instability of about 10^−15^ at 1 s. This allows us to narrow the VUV radiation linewidth substantially. Although we have not directly measured the VUV linewidth, spectral analysis suggests that it lies on the order of 10 kHz or less. In both cases, the scanning is provided by changing of the phase lock reference frequency.

Measurements of the absolute VUV laser frequency are performed by referencing to a signal from an active H-maser, traceable to Coordinated Universal Time (UTC), or an Yb^+^ single-ion clock^26^ established at the Austrian Federal Office of Metrology and Surveying (BEV), depending on the availability. The reference signal is delivered to the ATI by a Doppler-compensated fibre link. The optical scheme of the laser system is described in detail in Methods.

The detection chamber is designed to capture a large fraction of the fluorescence photons emitted by the Th:CaF_2_ crystal, as well as to monitor the power of the transmitted VUV laser. A schematic is shown in Fig. 1. The Th:CaF_2_ crystal is mounted on a three-axis vacuum translation stage used for precise alignment, such that the Cherenkov radiation emitted by the crystal^22^ and the transmitted VUV laser power is maximized. The fluorescence signal is spectrally resolved using a modified high-numerical-aperture Seya-Namioka spectrometer (HP Spectroscopy), in which an elliptical collection mirror with a diameter of 13.6 cm collects the emitted light from the Th:CaF_2_ crystal, which is placed in the first focus of the mirror and focuses it onto a 1-mm slit located at the seconds focus. After that, the light is diffracted and refocused by a spherical concave grating. Spatially resolved photon counting is performed by a CsI-coated microchannel plate (MCP) with a phosphor screen on the backside. The phosphorescence is finally detected using a complementary metal–oxide–semiconductor (CMOS) camera. Furthermore, a lead shield is used to protect the MCP from high-energy γ-radiation. For absorption measurements, laser alignment and VUV laser power measurements, a PMT with a CsI-coated photocathode (Hamamatsu R6835) is mounted behind the Th:CaF_2_ crystal.

## Fluorescence measurements

**Fig. 2:**
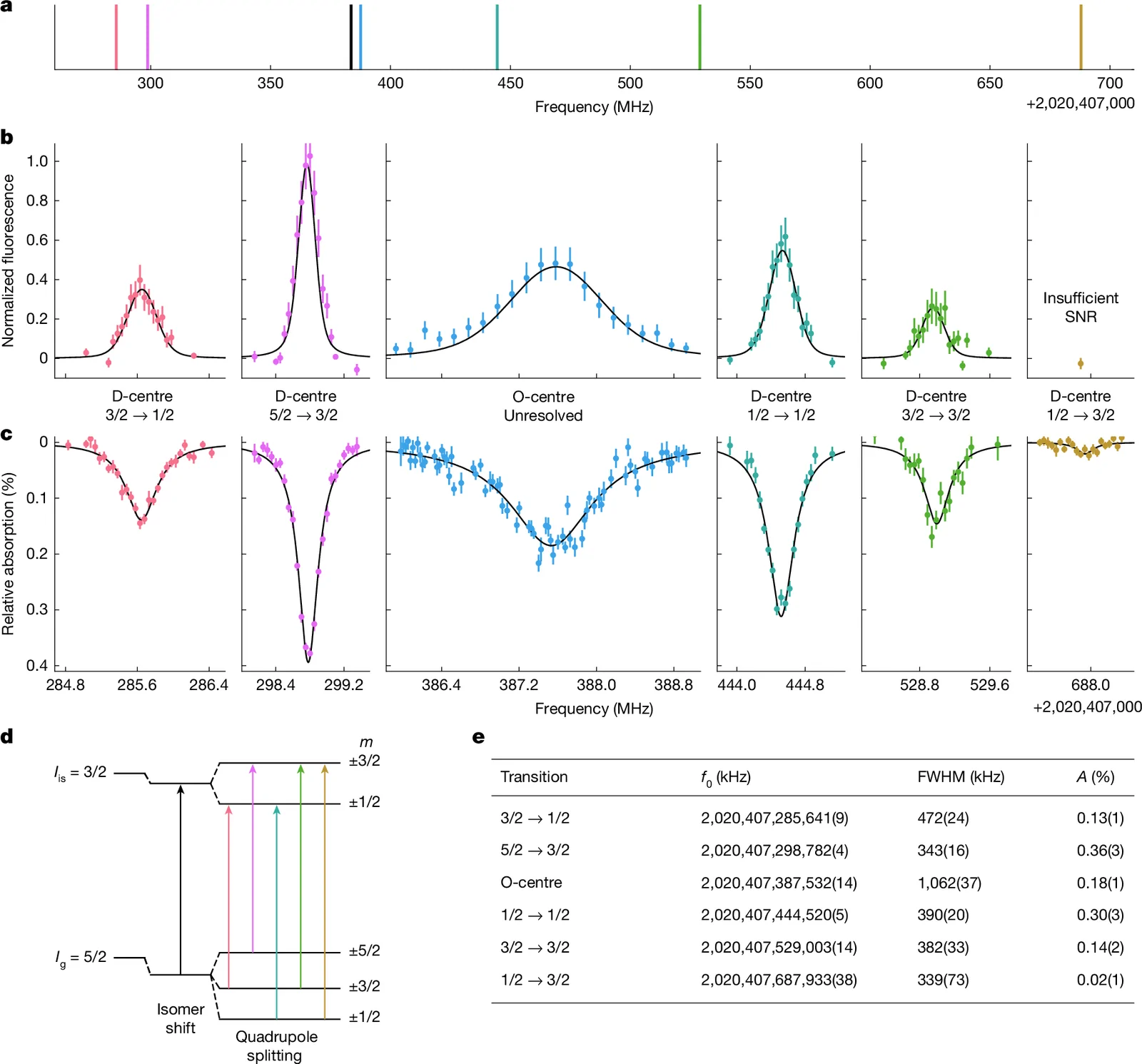
Fluorescence and absorption spectroscopy of the Th-229 nuclear transition, showing signals from two thorium centres (O and D). **a**, Overview of the detected lines on the MHz scale. The black line shows the calculated centre of the quadrupole-split D-centre. **b**, Fluorescence signals. All lines are normalized with the same factor. The 1/2 → 3/2 line was not measured, owing to low signal-to-noise ratio (SNR). Each frequency was measured two times; error bars indicate the Poissonian noise in the detected photon number. **c**, Absorption signals. The O-centre line corresponds to a thorium defect, which has a nearly vanishing EFG at the nucleus position. Error bars indicate the standard error of the mean. **d**, The Th-229 quadrupole structure level diagram of the D-centre including the isomer shift. The coloured arrows represent the observed transitions in **a**–**c**. **e**, Fitted spectral line parameters and relative transition strengths. Values of *f*_0_ correspond to the central frequencies of the individual transitions, FWHM corresponds to the full width at half maximum of the lines and *A* represents the absorption amplitude of the lines.

When performing spectroscopy with fluorescence detection, the Th:CaF_2_ crystal is periodically illuminated for 800 s, followed by a 800-s detection interval, dictated by the decay constant of the nuclear excited state. During detection, the laser radiation is blocked by a mechanical shutter to avoid any influence of stray light. In the beginning of the detection period, a fluorescence count rate of about 50 photons per second is observed, above a constant radioluminescence background of around 160 events per second. The VUV laser frequency is scanned in 50-kHz steps over the relevant frequency ranges of the quadrupole splitting in Th:CaF_2_ reported in refs. ^3,6^. We detect five lines shown in Fig. 2b, corresponding to two distinct Th-229 dopant sites in the crystal CaF_2_ structure (see Fig. 1 and discussion below). Each transition is scanned twice, once from lower to higher frequency and once from higher to lower frequency, to account for the asymmetry in the resonance curves owing to the long isomer decay time^1^. Figure 2b shows the corrected line profiles after superposition of data from both scan directions.

## Absorption measurements

In a new approach that is enabled by the narrow-linewidth CW laser, the transmission through the crystal is measured to record the absorption spectrum of the Th-229 nuclei. In contrast to fluorescence, this measurement can be performed continuously because the nuclear ground-state population is not markedly depleted and therefore the absorbed VUV laser power is approximately independent of time. The first absorption spectra shown in Fig. 2c are recorded with the 1,187-nm laser phase locked to the frequency comb mode and the PMT current is measured with a picoammeter.

The transmitted power is measured differentially and the absorption is calculated as (*I*_0_ − *I*)/*I*_0_, in which *I* is the detected laser intensity on-resonance and *I*_0_ is the detected intensity 2 MHz detuned off-resonance. This differential detection eliminates the influence of slow laser power fluctuations on the signal. A period of 4 s is used for alternating between the two frequencies. This measurement cycle is determined by the picoammeter readout. Each data point is averaged over 320 s. For the data shown in Fig. 2, the recording time in absorption is typically five times shorter than in fluorescence. The detected signal is around 6 × 10^6^ counts per second in photon counting mode and the noise is dominated by photon shot noise.

For a simple estimate of the expected photon absorption probability, we calculate *ρlλ*^2^*Γ*_eg_/(6πΔ*ω*) (ref. ^27^), in which *ρ* is the thorium concentration, *l* the crystal length, *λ* the nuclear transition wavelength and *Γ*_eg_/Δ*ω* is the ratio of the partial vacuum decay rate ($${\varGamma }_{{\rm{eg}}}\approx 2\pi \times 70\,\mu {\rm{Hz}}\times $$$${|{C}_{{I}_{{\rm{g}}}{m}_{{\rm{g}}}1q}^{{I}_{{\rm{e}}}{m}_{{\rm{e}}}}|}^{2}$$, in which $${C}_{{I}_{{\rm{g}}}{m}_{{\rm{g}}}1q}^{{I}_{{\rm{e}}}{m}_{{\rm{e}}}}$$ are Clebsch–Gordan coefficients, ranging between $$\sqrt{1/15}$$ and $$\sqrt{2/3}$$) and Δ*ω* ≈ 2π × 400 kHz is the broadened linewidth in the crystal. Taking *ρl* ≈ 3 × 10^22^ m^−2^, the estimate of the absorption probability ranges between 0.15% and 0.5%, depending on the specific transition. This is in reasonable agreement with the observed relative absorption if we take into account the distribution of thorium nuclei over different doping sites. Owing to this small cross-section, only a fraction of <10^−7^ nuclei are in the excited state at any time.

In absorption spectroscopy, we observe six lines owing to improved signal-to-noise ratio (Fig. 2c). Five lines are associated with a thorium dimer defect that has dihedral symmetry^6^ (D-centre; Fig. 2b) and was first reported in ref. ^3^. The fitted spectral parameters of the lines are presented in the table in Fig. 2e. Some of the measured line centres deviate by more than 3*σ* from previously reported values^24,28^, which we tentatively attribute to variations of the thorium concentration in the two segments of the X2 crystal used. The observed linewidths vary between 339 kHz and 472 kHz and are dominated by the laser linewidth in this set of measurements (Methods). We find Voigt line profiles to best describe the data in this regime. We fit the electric field gradient (EFG) and nuclear quadrupole moments using our determined line centres with the same method as in ref. ^28^ and find the best results for *Q*_*s*_*V*_*zz*_ = 335.32(2) eb V Å^−2^, *η* = 0.57183(9), ${Q}_{s}^{m}/{Q}_{s}=0.57005(2)$ and *f*_D_ = 2,020,407,383,542(3) kHz, in which *V*_*zz*_ is the largest entry in the diagonalized EFG matrix, by convention defining the *z* axis, *η* is the asymmetry of the EFG, ${Q}_{s}^{(m)}$ is the Th-229(m) spectroscopic quadrupole moment and *f*_D_ is the frequency of the D-centre transition after elimination of the quadrupole splitting.

We also observe a line close to the centre frequency of the quadrupole structure of the D-centre with a larger linewidth of about 1 MHz. This feature has already been reported in ref. ^6^, with a laser-limited linewidth of 30 MHz. It can be interpreted as the unresolved quadrupole structure of a thorium centre in a high-symmetry doping position in the crystal with nearly vanishing EFG. We use the name O-centre because of its O_h_ symmetry^29^.

**Fig. 3:**
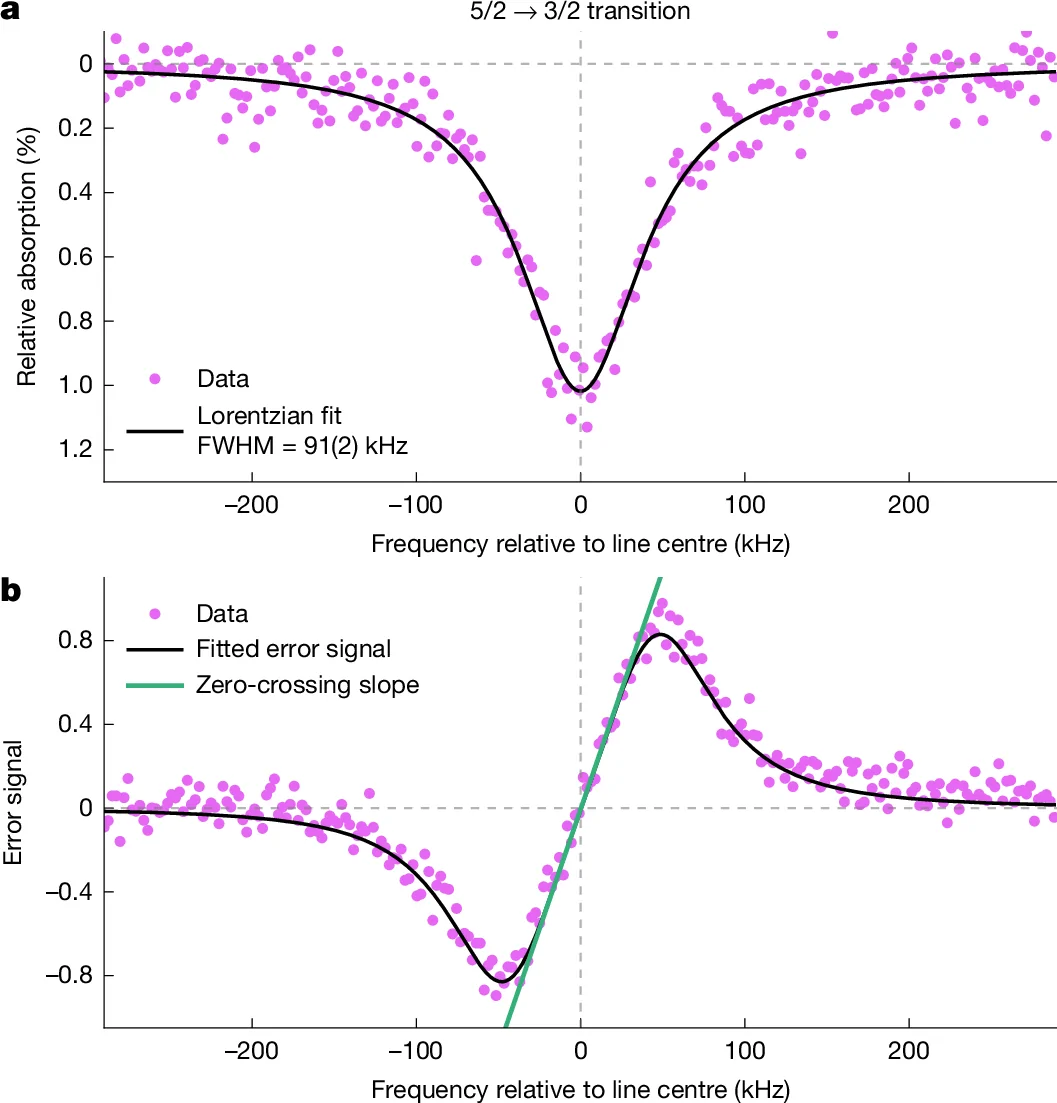
Absorption and error signals of the D-centre 5/2 → 3/2 transition observed with the narrow-linewidth cavity-stabilized VUV laser. **a**, The absorption signal of the 5/2 → 3/2 transition. **b**, The error signal of the absorption line is acquired at a modulation frequency of 10 Hz with the frequency deviation of 90 kHz. The signal slope is 2.28 × 10^−5^ Δ% Hz^−1^.

The operation of an optical clock requires a signal to stabilize a laser to the reference frequency. This signal can be conveniently produced by frequency-modulation spectroscopy that is capable of sensitive and rapid measurement of the absorption with narrow spectral features^13^. Here we demonstrate this method on a nuclear transition as a crucial step towards the operation of a nuclear clock. To reduce the VUV laser linewidth and noise level, the laser frequency was phase locked to an ECDL at 1,187 nm that is stabilized to a high-finesse cavity (Methods). The PMT readout was switched to photon counting mode, which allowed us to use a 10-Hz frequency modulation. Figure 3a shows the absorption profile of the 5/2 → 3/2 transition with 3-s averaging per data point. This constitutes an overall reduction of the detection cycle by two orders of magnitude compared with the fluorescence measurement. The spectral line has a full width at half maximum (FWHM) of 91(2) kHz, which is in agreement with the fluorescence signal width obtained in ref. ^10^ for the X2 crystal. We find spectra in which the laser linewidth is clearly below the nuclear transition linewidth to be best described by Lorentzian profiles. The detection of the absorption signal in the Th:CaF_2_ crystal allows us to record an error signal with zero-crossing at the resonance frequency, observed by first-harmonic detection (Fig. 3b). The error signal is acquired with a frequency deviation of 90 kHz and averaging the signal over the same integration time as for the absorption profile shown in Fig. 3a.

**Fig. 4:**
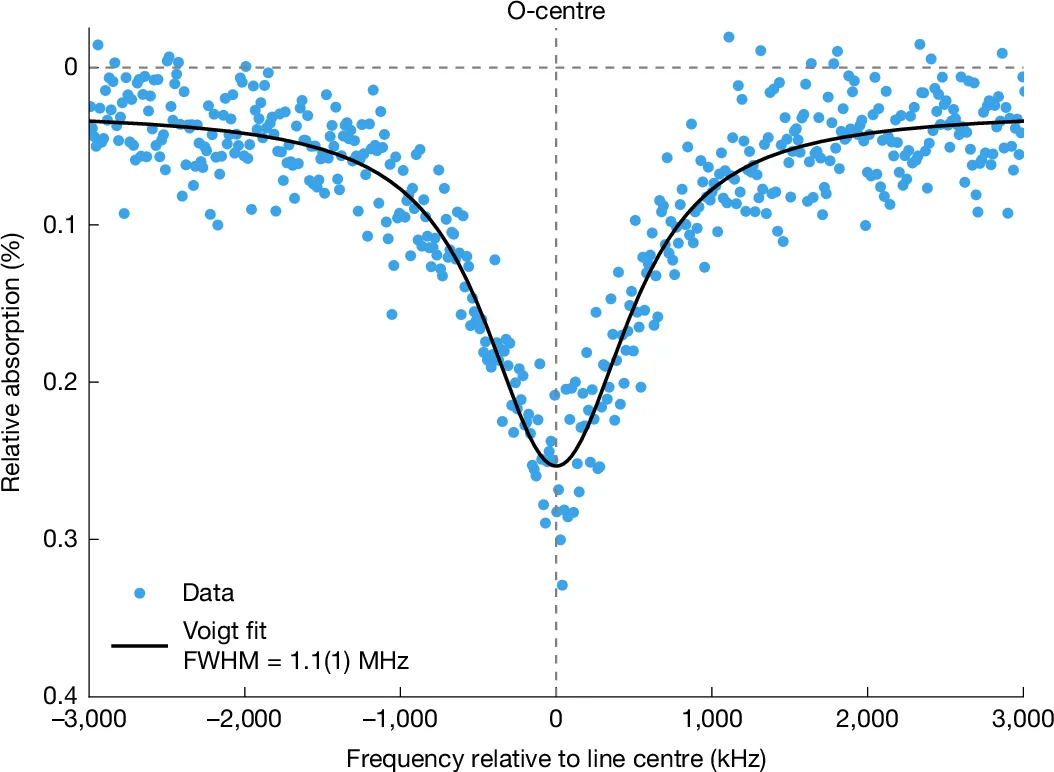
Absorption signal of the O-centre line. The absorption signal is recorded with the narrow-linewidth cavity-stabilized VUV laser, similar to the D-centre line shown in Fig. 3. No substructure is resolved.

The absorption resonance of the O-centre recorded with the narrow-linewidth laser is shown in Fig. 4. For the X2 crystal used in this experiment, we do not observe a resolved quadrupole spectrum for this centre. From the linewidth and symmetry of the O-centre resonance, we can derive an upper bound on the absolute value of the EFG’s *V*_*zz*_ component of <0.1 V Å^−2^ compared with roughly 100 V Å^−2^ for the D-centre^3,6^ (Methods). The differences in line broadening observed between the D-centre and the O-centre will be a subject of further investigations.

## Isomeric shift

The O-centre line is offset by Δ*f*_OD_ = *f*_O_ − *f*_D_ = 3.99(2) MHz from the centre of the quadrupole structure of the D-centre (black line in Fig. 2a) determined in the previous section, which we attribute to a difference in the isomer shift^30^. The difference in the isomer shifts indicates that the electron density at the thorium nucleus is higher for the O-centre than for the D-centre. The shift agrees with the results of density functional theory (DFT) simulations for the D-centre and O-centre (Methods), further corroborating the assignment.

## Discussion and outlook

We demonstrate the first precision spectroscopy of the Th-229 nuclear resonance of the O-centre and revalidate the proposed microscopic doping structures of the O-centre and D-centre by showing agreement between the measured and calculated isomer shift. The O-centre line was not reported in laser excitation with a VUV frequency comb^3,10,24^ but it was observed using a pulsed VUV FWM source^6^ and in the present experiment. We conjecture that this difference might be related to light-induced quenching^17,18,20^ through non-resonant modes in VUV frequency combs.

Further high-resolution spectroscopy of the O-centre and other known centres^6^ can benchmark theoretical models for thorium doping in CaF_2_, further improving on the isomer shift and EFG calculations. Using the O-centre for a solid-state nuclear clock should be further investigated with respect to benefits over using the D-centre, especially concerning a possibly increased robustness against lattice deformations. Working with smaller thorium doping concentration has led to lower linewidths of approximately 30 kHz in the case of the D-centre, attributed to a lower level of microstrain^10^. It will be critical to see whether the individual components of the underlying quadrupole structure can be resolved, as for the D-centre^10^. Measuring the temperature-induced frequency shifts and broadening effects will further contribute to the characterization of the O-centre.

Most importantly, we demonstrate the first detection of an absorption signal of the Th-229 nuclear transition. Previous experiments based on detection of fluorescence light were limited by the long decay time of the metastable state. Absorption spectroscopy, by contrast, measures the excitation in real time and is therefore more suitable for stabilizing a laser on the thorium transition.

In the future, absorption spectroscopy will enable the realization of a nuclear clock with fast feedback cycle^31,32^. On the basis of the measured linewidth of Δ*ν* ≈ 100 kHz for the 5/2 → 3/2 component of the D-centre, a detected photon flux ${\dot{N}}_{\gamma }\approx 6\times 1{0}^{6}\,{{\rm{s}}}^{-1}$ and the absorption fraction *A* ≈ 0.01, the present set-up suggests a fractional frequency instability of $\sigma (\tau )\approx \Delta \nu /({\nu }_{0}\,A{({\dot{N}}_{\gamma }\tau )}^{1/2})\approx 2\times 1{0}^{-12}\sqrt{\tau \,({\rm{s}})}$, in which *τ* is the averaging time and assuming a shot-noise-limited measurement.

Further improvements in linewidth, detected photon flux and absorption fraction can be achieved by several means. The interaction length *l* can be increased by using longer crystals or by integration with an optical cavity. The CW laser power *I*_0_ can be increased by using different nonlinear media for the generation of VUV^11^ or cavity-enhanced SHG in nonlinear crystals such as SBO or BaMgF_4_ (BMF). For the present laser source based on SBO^5^, we expect a roughly tenfold increase in VUV power for cavity-enhanced SHG. Crystalline host materials different from CaF_2_, which should show different scaling of nuclear linewidths with concentration, can be explored. A main benefit of a thorium solid-state clock lies in its potential long-term stability, using a similar approach to optical frequency standards based on resonances in ions or neutral atoms. All of the above-mentioned factors together can result in more than two orders improved shot-noise-limited clock performance. Therefore, we can expect to reach an instability of ≤10^−16^ at about 10^4^ s with further improvement at long timescales.

In conclusion, in this work, we overcome the limitation of the fluorescence detection method caused by the long radiative decay time of the isomeric state of Th-229. Absorption spectroscopy with a CW VUV laser source provides a clear pathway to operating a solid-state nuclear clock of high stability and accuracy.

## Methods

### Experimental apparatus and VUV beam guiding

**Extended Data Fig. 1:**
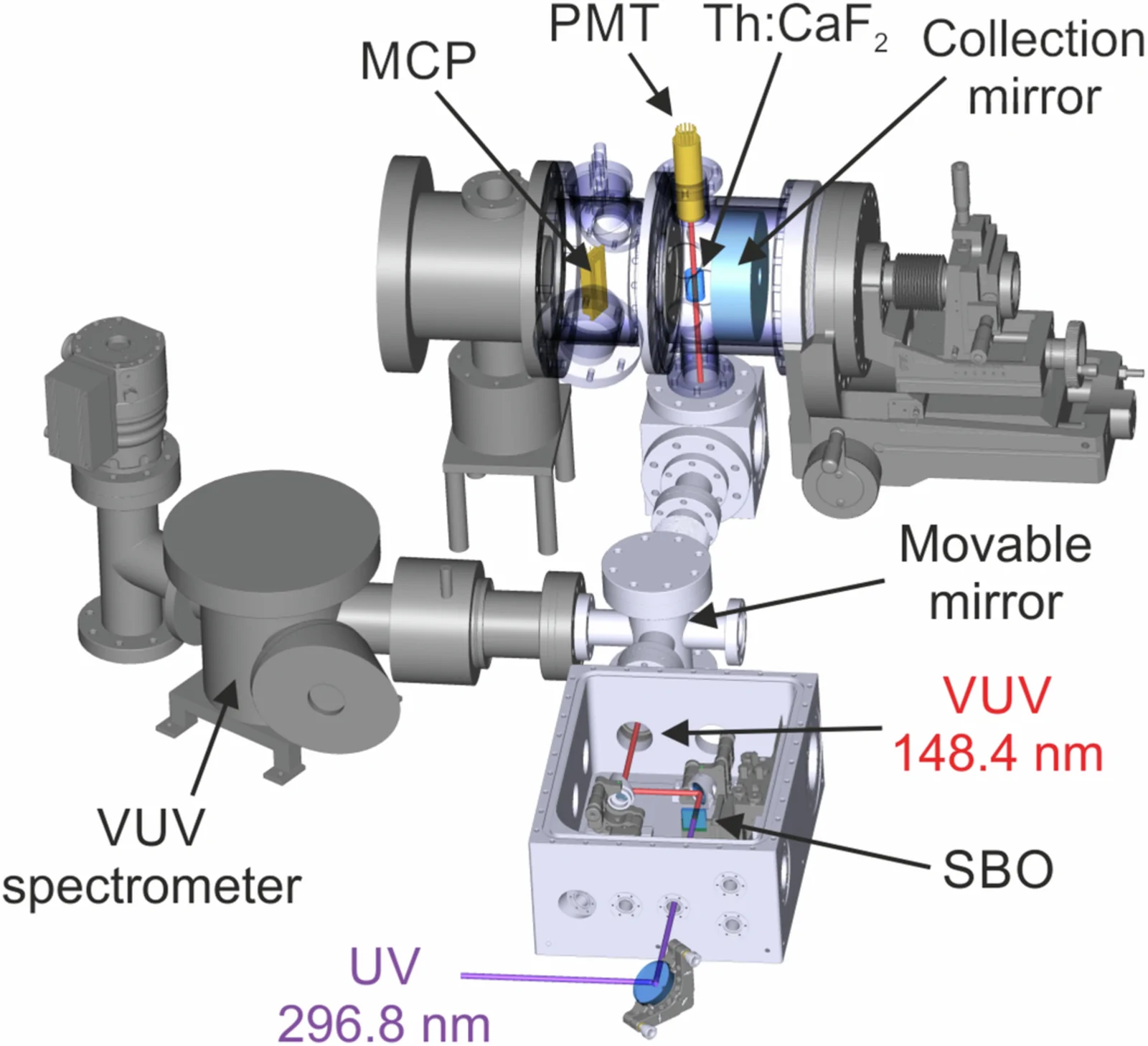
The experimental apparatus. The VUV light is generated by a single-pass SHG in a random quasi-phase-matched SBO crystal and directed to the thorium-doped crystal. The VUV spectrometer is used for optimization and diagnostics of the VUV laser.

The design of the spectroscopy apparatus is shown in Extended Data Fig. 1. It consists of the chamber with the SBO crystal for SHG frequency conversion, a vacuum beamline, a VUV spectrometer and a spectroscopy chamber with a thorium-doped crystal and the detection system. The generated VUV beam is aligned to the Th-229 crystal by one plane and two curved dichroic mirrors mounted on motorized mounts using ultraviolet radiation as a pilot beam. The incidence angle of the radiation on all of the mirrors is 45°. Each dichroic mirror has approximately 90% reflectivity at 148.4 nm and approximately 99% transmission for ultraviolet at this angle and therefore separate the generated VUV beam from unconverted 296.8-nm radiation. The CsI PMT has a quantum efficiency of ≤10^−5^ in ultraviolet. Therefore, the remaining ultraviolet background signal registered on the PMT does not exceed the 5% level of the VUV power transmitted through the crystal. A CMOS camera is used for the initial alignment of the pilot beam through the crystal in the spectroscopy chamber. To switch between paths that guide to either the VUV spectrometer for power measurements or the spectroscopy chamber for crystal experiments, the vacuum beamline is equipped with a movable mirror.

### Laser frequency stabilization and scanning

**Extended Data Fig. 2:**
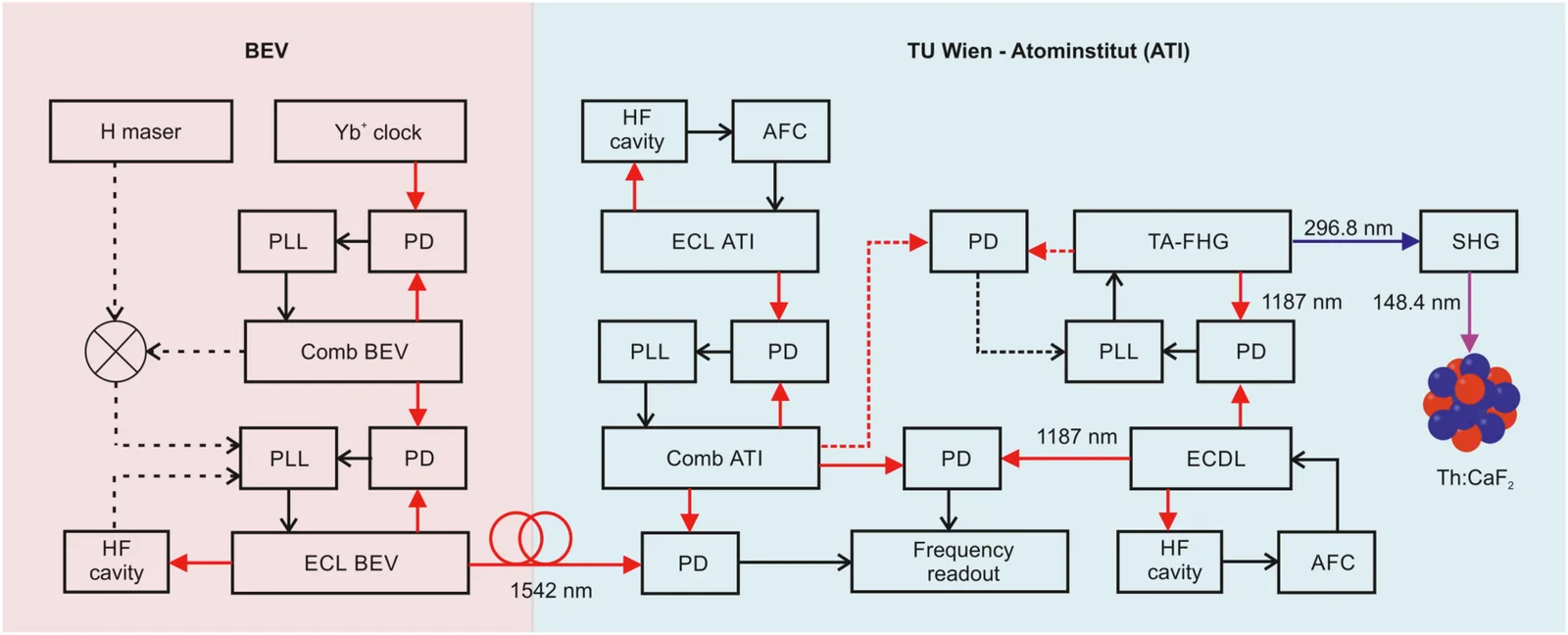
Optical set-up and locking scheme used in our experiment. Frequency measurements are performed by referencing to a signal from BEV, traceable to either UTC or an Yb^+^ single-ion clock. The TA-FHG laser is phase locked either to a relevant comb mode or to the cavity-stabilized ECDL at 1,187 nm.

The laser arrangement used for the spectroscopy experiments is shown in Extended Data Fig. 2. The frequency comb located at BEV based on an erbium-doped fibre laser is fully stabilized by locking the carrier-envelope offset frequency to a radiofrequency reference and by locking the repetition rate to either an external cavity laser at 1,542 nm (ECL BEV) dedrifted with a feedback loop to an active H-maser traceable to UTC or to an Yb^+^ single-ion clock^26^. In case the Yb^+^ clock is used as reference, the radiation frequency of the ECL BEV is stabilized by locking to a relevant comb mode. The ECL BEV is then guided through a length-stabilized optical fibre link to ATI. The second optical comb located at ATI is also fully stabilized by locking to a high-finesse cavity-stabilized fibre laser and a radiofrequency reference. The long-term drift of the comb repetition rate is compensated by analysing a beat signal with the ECL BEV. Frequency stabilization and scanning of the high-power laser system at 296.8 nm are provided by a phase lock loop (PLL) of its radiation to a relevant ATI comb mode (dashed lines) or by a phase lock to the radiation of a frequency-stabilized ECDL at 1,187 nm. The ECDL is frequency-stabilized by an automatic frequency control locking system. In both cases, the scanning is provided by changing of the PLL reference frequency. The frequency chain has a systematic frequency uncertainty of ±1 kHz in the VUV.

The nuclear transitions’ FWHM spectral width of ≳300 kHz observed by locking of the high-power 1,187-nm laser directly to a comb mode is similar to the value reported in ref. ^3^ and is most probably limited by phase noise of the reference comb mode transferred by the PLL to the upconverted light linewidth. Therefore, the VUV linewidth in this operation mode is on the order of 300 kHz.

For the high-power laser phase locked to the high-finesse cavity-stabilized ECDL, we detected a 91(2)-kHz spectral FWHM of the 5/2 → 3/2 transition for the X2 crystal, in agreement with ref. ^10^. Although we did not directly measure the laser linewidth in VUV, we give here only our estimations based on the width of detected spectroscopy signals. The actual laser linewidth is expected to be ≤10 kHz assuming the same transition spectral width observed in earlier experiments using an optical comb^10^ with about 1-kHz VUV linewidth.

### O-centre linewidth estimation

The linewidth of the O-centre of 1.1(1) MHz (Fig. 4) clearly exceeds the laser linewidth in all measurements. We have verified, by performing broadband scans, that it is not a single transition belonging to a quadrupole structure of another thorium defect centre in the crystal, analogous to previous work in ref. ^6^. We conjecture that it is the unresolved quadrupole structure of a thorium centre in a high-symmetry dopant site with small or fully vanishing EFG. To constrain the maximum static EFG in terms of *V*_*zz*_, we have fitted the O-centre line with a set of six quadrupole transitions with the respective transition strengths^28^. For modelling, we assume a Cauchy–Lorentz distributed EFG, characterized by a distribution width *δV*_*zz*_ (half width at half maximum) and a distribution centre *V*_*zz*_, which describe EFG fluctuations and the static field gradient contribution, respectively. We justify this distribution based on the scaling behaviour of the EFG (*V*_*zz*_ ∝ *r*^−3^) and by assuming that independently distributed point defects induce fluctuations in the EFG at the thorium site, at which the probability of such a point defect being located in a spherical shell with width d*r* is *p*(*r*)d*r* ∝ *r*^2^d*r*. Changing *r* to *V*_*zz*_ yields the probability $p({V}_{{zz}}){\rm{d}}{V}_{{zz}}\propto {V}_{{zz}}^{-2}{\rm{d}}{V}_{{zz}}$. The Cauchy–Lorentz distribution provides the correct scaling at its tails and, also, is symmetric about the origin. Although the Cauchy–Lorentz distribution has a formally undefined mean, this does not affect the physically relevant, fitted quantities *V*_*zz*_ and *δV*_*zz*_, which remain well defined regardless of this property^33^. In our fitting procedure, the *η* parameter was fixed to 0.57 (D-centre value) and we obtain *δV*_*zz*_ = 0.928(1) V Å^−2^ and *V*_*zz*_ = 0.02(4) V Å^−2^, with the errors being extracted from the relevant elements of the covariance matrix. An upper bound of *δV*_*zz*_ = 0.1 V Å^−^^2^ also holds when fixing *η* to either end 0 or 1. The very low *V*_*zz*_ value indicates a nucleus in a defect centre with O_h_ symmetry. The fluctuations in *δV*_*zz*_ exceed those found for the D-centre in a similar analysis by a factor of about ten. We note that the fluctuations introduced by *δV*_*zz*_ constitute a generic model for inhomogeneous broadening, allowing us to compare different defect centres, whereas the underlying physical mechanisms governing the observed linewidths of the D-centre and O-centre (and their observed concentration dependence) remain to be identified.

### Calculations of isomer shifts using DFT

To numerically simulate the defect centre properties using DFT, we use a two-step procedure. First we construct defect centres in a 2 × 2 × 2 supercell of the conventional CaF_2_ unit cell by replacing one or two (adjacent) Ca ions by Th ions, respectively. We then relax these initial ionic positions to minimize the energy of the system. At several steps along the relaxation trajectory, we repeatedly optimize the supercell lattice vectors. After the relaxation criteria are met, we verify that the relaxed structures do not contain imaginary phonon frequencies.

We performed the calculations of the first step using the plane-wave Vienna Ab initio Simulation Package (VASP)^34–38^ with an energy cut-off of 800 eV at the Γ-point. Our convergence criterion of forces imposed a maximum absolute value of 0.00001 eV Å^−1^ on the largest ionic force, whereas we stopped volume optimizations when the energy difference between subsequent iterations was less than 10^−7^ eV. We used the phonopy software^39,40^ to compute phonon band structures. The resulting calculations revealed that neither structure exhibited states with imaginary frequencies, apart from inherent numerical inaccuracies. As a result, we concluded that our simulations had converged to the global structural minimum.

In the second step, we calculated the isomer shift for thorium using the electron density difference at the smallest grid point within the linearized-augmented-plane-wave basis in the WIEN2k code^41^. For these calculations, we used the -prec 2n setting and our convergence criterion was a change in the electronic charge density of less than 0.00001 Rydberg atomic units, as specified by the -cc 0.00001 option. We performed convergence tests to the -prec 1n setting and found changes in the isomer shift of about 1 MHz. We used the Perdew–Burke–Ernzerhof approximation^42^ for the exchange-correlation potential throughout all calculations.

The isomeric shift between two electronic environments A and B is^43^

1 $$\Delta {E}_{{\rm{AB}}}=\frac{{eZ}}{6{\varepsilon }_{0}}\Delta {\rho }_{{\rm{AB}}}\Delta \langle {R}^{2}\rangle ,$$

in which *ε*_0_ is the vacuum permeability, *e* is the elementary charge, *Z* = 90 is the nuclear charge and $\Delta \langle {R}^{2}\rangle =\langle {R}_{{\rm{m}}}^{2}\rangle -\langle {R}_{{\rm{g}}}^{2}\rangle =0.0107(9)\,{{\rm{fm}}}^{2}$ for Th-229, being the average of three reference values^28,44,45^. The electronic environment A corresponds to the single thorium high-symmetry O-centre and environment B is the dimer-like D-centre.

From our simulations, we obtained values of the electronic charge density difference between the O-centre and D-centre $$\Delta {\rho }_{{\rm{OD}}}=$$$$2.436829687\times 1{0}^{-5}\,e/{r}_{{\rm{Th}}}^{3}-2.436829589\times 1{0}^{-5}\,e/{r}_{{\rm{Th}}}^{3}=9.755\times 1{0}^{-13}$$$$e/{r}_{{\rm{Th}}}^{3}$$, in which *r*_Th_ = 5.7557 fm denotes the radius of the Th-229 ground state^46^. We calculated the corresponding energy shift Δ*E*_OD_ = 88.0698614 THz − 88.0698579 THz = +3.60(29) MHz. We determine the experimental reference by subtracting the field-free frequency of the D-centre from the central line frequency *f*_0_ of the O-centre as +3.99(2) MHz. We propose that the coexistence of isolated thorium and thorium dimers reflects the stochastic distribution of dopants during crystal growth. At higher doping concentrations, dimer formation becomes more probable as the average inter-dopant spacing decreases.

**Extended Data Fig. 3:**
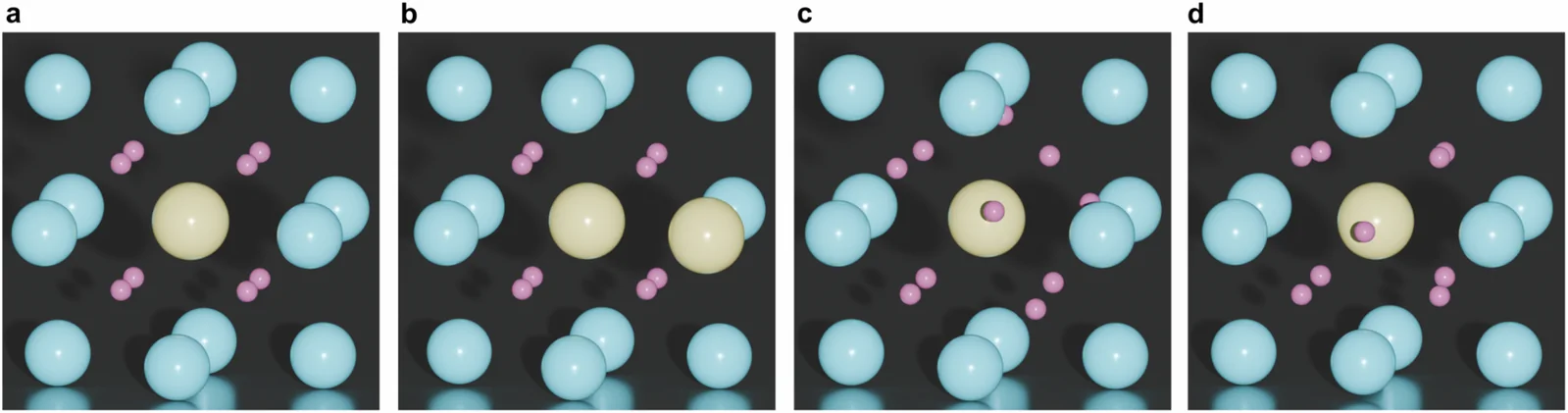
Relaxed structures of the investigated defects in this study. The calcium atoms are represented in blue, fluorine atoms in pink and thorium atoms in yellow. **a**–**d**, The panels show: the O-centre (O_h_ defect point group symmetry) (**a**); the D-centre (D_2h_) (**b**); two added F ions (C_3v_) (**c**); and a single F interstitial (C_4v_) (**d**).

Before the assignment of the observed O-centre and D-centre based on their respective EFGs performed in ref. ^6^, thorium dopant geometries involving local charge compensation through interstitial F ions were discussed^47,48^ (see Extended Data Fig. 3 for all investigated defect structures). Initial placement of two interstitial fluorine atoms relaxes the lattice into a C_3v_ point-group symmetry around the defect^48^. A defect centre involving a single interstitial fluorine and singly charged by removing an electron (to preserve closed shells) remains in a C_4v_ symmetry. Although the calculated EFGs for these centres (respectively, *V*_*zz*_ = −68 V Å^−2^, *V*_*zz*_ = −279 V Å^−2^ and *η* = 0 for both) do not match the observed values, we report their isomer shifts for comparison purposes. The simulations yield an isomer shift of $\Delta {E}_{{{\rm{C}}}_{3{\rm{v}}}{\rm{D}}}=0.076(1)\,{\rm{MHz}}$, whereas $\Delta {E}_{{{\rm{C}}}_{4{\rm{v}}}{\rm{D}}}=-32(3)\,{\rm{MHz}}$.

## Online content

Any methods, additional references, Nature Portfolio reporting summaries, source data, extended data, supplementary information, acknowledgements, peer review information; details of author contributions and competing interests; and statements of data and code availability are available at https://doi.org/10.1038/s41586-026-11011-7.

## Supplementary information

Peer Review File

## Data Availability

The data that support the findings of this study are available from the corresponding authors on request.
