# Supplementary material for: Continuous-wave laser absorption spectroscopy of the thorium-229 nucleus
Source: Nature. 2026 Sep 16;657(8132):626–31. doi: 10.1038/s41586-026-11011-7 (PMC13581605; doi:10.1038/s41586-026-11011-7)
Supplement: Supplementary file 1 [file 41586_2026_11011_MOESM1_ESM.pdf]

nature portfolio

Peer Review File

Continuous-wave laser absorption spectroscopy of the Thorium-229 nucleus

Corresponding Author: Professor Thorsten Schumm

Any redactions in this file are there to maintain patient confidentiality, the confidentiality of unpublished data, or to remove third-party material.

This file contains all reviewer reports in order by version, followed by all author rebuttals in order by version.

Version 0:

Reviewer comments:

Referee #1

(Remarks to the Author)
Report on Nature ms 2026-04-10959 by Morawetz et al.

This manuscript reports on advances in laser spectroscopic studies of the by now famous thorium isomer in Th-229. This isomer is seen as a key component for constructing an ultraprecise “nuclear clock” that potentially outperforms the best atomic clocks. It is based on the unique nuclear system Th-229/Th-229m, where the latter is the lowest-lying known nuclear isomer, situated at an excitation energy of only 8.4 eV, accessible by VUV laser systems. The clock concept pursued in the present manuscript is based on embedding Th-229 in a VUV-transparent large band-gap host material, which preserves the Th in ionic state in the lattice, which is necessary to energetically close the fast internal-conversion decay channel open to the isomer in neutral atoms. In the absence of an open non-radiative deexcitation channel, slow radiative deexcitation takes place, on a timescale of hundreds of seconds. Photon detection from this decay mode (nuclear fluorescence) was achieved in 2023 (Kraemer et al., Nature 2023), followed by direct laser excitation of the isomer (refs. [1-3] in the present manuscript). The long radiative lifetime of the Th-229m ionic system makes the cycling process needed for clock operation slow, as a next excitation cycle can only start after a substantial fraction of isomers has deexcited; this translates to a small duty cycle available for comparison of an oscillator to the nuclear clock reference, thus preventing the exploitation of the full power of the Th-229 system.
In the present manuscript, the authors describe experiments in which they overcome the limitation of the slow photonic decay of Th-229m. Instead of recording photons emitted in the state’s decay, they record the absorption of laser photons due to successful excitation from the ground to the isomer state within the Th-doped crystal. A significant effect can only be seen if the laser light is quantitatively contained inside the absorption linewidth of the nuclear transition. This technique provides almost immediate response; in their work, the authors used a 3 s cycling time, which is an improvement of at least two orders of magnitude, but this is not yet at the physics limit. In addition, detailed information on the lattice sites of Th in the used SBO crystal is obtained as well as information on quadrupole splitting of the optical lines at the two sites, where one shows a small static electrical field gradient, which is optimal for the construction of an environment-insensitive clock.

The achievement and successful demonstration of absorption spectroscopy and its implications for nuclear clock operation make this manuscript suitable for Nature, and hence we recommend to accept the paper after the authors take the following comments into account:

Title: the title suggests a cw laser to be a necessary prerequisite for the presented work, and it also suggests that this is a new achievement. In its current form, the title even starts with “cw nuclear laser”, which is misleading. For us, the essence of the work is not the continuous-wave laser (which was already published by the group in their ref. [5], and where another group operates a stronger cw laser, see ref. [11] in the present manuscript) but establishing absorption spectroscopy to allow for substantially shorter cycling times in state-interrogation. The key is in the narrow linewidth, which requires a cw laser. A suggestion for the title is “Narrow bandwidth laser absorption spectroscopy in the nuclear isomer transition of thorium-229”.

Abstract: in line with the above, the statement on line 7 on “continuous wave” should state “narrow bandwidth”, i.e., “can be excited with a continuous-wave narrow bandwidth laser source”. The abstract closes with the statement “…, promising

nuclear resonance lines that are less sensitive to the lattice spacing". In a broad journal like Nature it might be appropriate to state explicitly why this is advantageous. Also, the question of "less sensitive than what" lingers. One could replace "less sensitive" by "quite independent" in this last sentence.

Body text:

Page 2:
the natural linewidth is first given as being in the millihertz range, and then as being crystal-field-broadened to 10-100 kHz. The authors then write that exploiting the full potential of a Th-229 solid-state nuclear clock requires a laser source with a linewidth that is smaller than the nuclear linewidth, but it is left open which of the two values stated earlier this refers to. It follows implicitly from the text on page 3, but it would be helpful at this point if this were stated more explicitly. Also, as far as the quoted millihertz linewidth goes, it should be added that this is theoretically predicted (including a reference) but not experimentally determined to date. In addition, we consider "smaller than the nuclear linewidth" too strict a requirement; "the same order" would appear more fitting to us. Finally, a reference should also be added for the 10-100 kHz crystal-field-broadened linewidth.
-For the non-expert reader it might be helpful to add a side remark of why the requirement of a narrow linewidth is only fulfilled in continuous wave lasers, but not in pulsed ones. Fundamentally, the narrow linewidth is the physics requirement.
-"in the VUV and possess" -> "in the VUV spectral region and possess"

Page 3:
-the first text line states that the laser linewidth can be narrowed, but there is no quantitative statement to which linewidth this is possible. This should be added.
-the sentence "A spectroscopic signal can be recorded by modulating the laser frequency over a fraction of the absorption linewidth…" likely refers to Pound-Drever-Hall-locking, which should be added.
-"The ability to perform absorption detection is pivotal for the operation of a solid-state nuclear clock. It probes…": does the "It" starting the next sentence refer to the absorption detection or to the operation of a clock"?
- the authors should add a sentence explaining why the coherence time in the crystal is so short. Is it the influence by the lattice, and is there a citation available for this?
-"It minimizes the detrimental effects on the signal from non-resonant VUV photons. These may…": These -> Effects. Otherwise the "these" refers to the photons.

Page 4:
-How was the optical transmission of the crystal at the nuclear transition wavelength measured to be 40%? By the absorption spectroscopy presented in the paper? What is the precision of this value?
Fig. 1: (1) this figure is almost impossible to comprehend for non-specialists, not least as absorption and fluorescence spectroscopy are not clearly separated. Also, the crystal, which is at the center of the experiment, is barely visible; (2) the component deflecting the green phosphorescence is not labeled; (3) the slit is drawn in a manner as if this and the MCP formed a single part. The figure is ideally redrawn with a clearer separation of all components; (4) the role of the slit is not mentioned in the caption; (5) the fluorescence appears to be drawn including unphysical angles; it is not evident that the mirror emits the phosphorescence that is eventually detected by the camera; (6) in b/w printouts, the O-center and D-center zoom regions appear exactly identical. The authors should select colors with different gray tones for Ca and Th-229; (7) why is there a central Th atom indicated in the center of the cell of the D center inset? (8) there should be consistence in the abbreviations in the figure and in the caption.

Page 5:
-the top part of the page describes a first laser generating 1187 nm light, which is frequency quadrupled to 296.8 nm, after which frequency doubling to 148.4 nm is done. Later on, separation of the desired 148.4 nm light from "fundamental UV" radiation is performed. However, "fundamental" is the light at 1187 nm, not at 296.8 nm.
-it would be useful to discuss the width of the fundamental linewidth and the increase in width due to the different doubling mechanisms.
-define UTC(BEV)
-is the "elliptical mirror" described in the text identical to the "collection mirror" shown in Fig. 1? If so, identical nomenclature should be used.

Page 6:
-The authors state that "the frequency is stepwise shifted by 2 MHz" in the absorption measurements. In connection with Fig. 2, however, where the text states that the Fig. shows fluorescence and absorption measurements, there are spectra shown with much smaller step size, tracing the linewidths on the level of less than 100 kHz. So other steps were used as well. The authors should also write explicitly to panel b of Fig. 2 that this shows fluorescence measurements. It can be inferred from the sign of the signal, and in panel c it can also be inferred from the y-axis label that these are the absorption measurements, but explicit statements are helpful especially for non-specialists. It would also be interesting to learn what the linewidths are, which seem surprisingly similar in both spectroscopy methods.
-the expression sqrt(1/15) – sqrt(2/3) reads as a mathematical expression, i.e., a subtraction, but it means a range, i.e. sqrt(1/15) to sqrt(2/3)
-"…the estimate of the absorption probability is of 0.006 GAMMA eg/GAMMA…" -> is of the order of?
-after "In absorption spectroscopy, we observe six lines" the authors could add "in contrast to fluorescence spectroscopy where only five are observed.

Page 7:

-the authors note that some of their reported line centers deviate from literature by more than 3 sigma, and give a tentative explanation. This suggests that similar fluctuations will occur in future studies using different crystals. What are the implications of this fact for stable clock operation?
-what is the minimum laser linewidth the authors have achieved?

Page 8:
-in Fig. 2 a, the panel should be divided in vertical direction into an upper panel for the O center and a lower panel for the D center. In the current form it is very hard to understand.
-the center of the quadrupole-structure of the D center is once given as "the black line" in panel Fig. 2d and as "the black dotted line" in Fig. 2a. Ideally, the same is used in both panels, and in the text, and ideally as solid line.
-in between Fig. 2b and Fig. 2c, the nomenclature "3/2 -> 1/2" on the one hand and "O-center" on the other hand is used. We suggest to add "D-center 3/2 -> 1/2" etc. space permitting. Or, even better, the panels of the O center should be separated from the panels of the D center.
-the arrangement of the frequency labels on top of Fig. 2a is not helpful. It leads to an inferred correspondence of the frequencies of panel a with panels b and c, which is not the case. The labels should be given below panel a to avoid this.

Page 9:
-In the second sentence in the Discussion part, the authors refer to "This line", but it is not explicitly stated which one this is. The authors then express their conjecture on the presence vs. absence of said line, but the reader cannot get any information on the implications on this. A sentence or two on this would be helpful to qualify the relevance of this discrepancy among different studies.

Page 10:
-Fig. 4 is not necessary and can be omitted.

signed Prof. Christoph Düllmann and Prof. Klaus Wendt

Referee #2

(Remarks to the Author)
This manuscript reports a milestone advance in the field of 229Th solid-state nuclear clocks. For the first time, it achieves continuous-wave laser absorption spectroscopy of nuclear transitions, breaking through the core bottleneck of traditional fluorescence detection limited by the ~600 s long lifetime of the nuclear excited state. Through high-resolution absorption spectroscopy, it resolves the nuclear transition properties of the D-center and O-center in CaF_2 crystals, and discovers that the O-center has an extremely low static electric field gradient of < 0.1 V/Å², laying a core technical foundation for the practical application of solid-state nuclear clocks. However, the manuscript still has the following issues that require revision and supplementation by the authors.

(1) The manuscript assigns the O-center to an O_h high-symmetry configuration. Theoretically, the electrostatic field gradient at the nuclear site under this symmetry is spherically symmetric, and the EFG asymmetry parameter η should be strictly zero. However, when fitting the EFG of the O-center, the paper fixes η to 0.57 (the value of the D-center) and only states that η has no substantive effect on the results. This assumption directly contradicts the high-symmetry property of the O-center, which may introduce systematic bias in the estimation of the upper bound of Vzz and undermine the core conclusion of the extremely low EFG of the O-center.

(2) The observed absorption signal amplitude is only 0.02%–0.36%, which is an extremely weak signal. However, no control experiments have been conducted to exclude non-nuclear transition contributions. It is impossible to completely rule out interference from non-nuclear factors such as crystal color centers, impurity absorption, laser-induced transient defects, and background fluctuations of Cherenkov radiation.

(3) The manuscript only provides an estimated fractional frequency instability of 2×10^{-12} at 1 s averaging time based on the shot-noise limit, but lacks quantitative noise analysis and long-term stability data. It is impossible to determine the current noise-limiting factor (shot noise / technical noise) of the system, nor to evaluate the reliability of absorption detection in the long-term operation of nuclear clocks.

(4) All experiments were performed at a single temperature of 294.7(5) K, and data on the dependence of nuclear transition frequency, linewidth, and absorption amplitude on temperature are missing. The temperature coefficient is the most important source of systematic error for solid-state nuclear clocks and directly determines the required temperature control precision of the clock. It is recommended to perform a systematic characterization of the effect of temperature on the spectral and frequency performance of the crystals.

(5) All experiments were performed on a single crystal with a bulk 229Th concentration of 6.6×10^{15} mm^{-3}, and no quantitative correlation data between concentration and the O/D center ratio, linewidth, absorption amplitude, and signal-to-noise ratio are provided. It is impossible to determine the optimal doping concentration for nuclear clocks, nor to verify whether reducing the concentration can narrow the linewidth of the O-center and resolve its quadrupole structure. The authors are suggested to supplement the study on the effect of 229Th doping concentration on crystal performance.

(6) The theoretical isomer shift (3.526 MHz) is in good agreement with the experimental value (3.991±0.013 MHz), but the calculation relies on the literature value $\Delta\langle R^2\rangle$=0.0105(13) fm², and its 12% relative uncertainty has not been quantified. In addition, independent verification methods for the electron density difference using other experimental techniques are not discussed. The formation conditions of the O-center and the coexistence mechanism with thorium clusters also remain unclear.

(7) It is well known that thorium clusters (such as the D-center) are common in Th:CaF_2, but the manuscript does not clarify the stable formation conditions and charge compensation mechanism of the O-center.

Referee #3

(Remarks to the Author)
This work describes the first spectroscopy of the nuclear transition in 229 thorium by a continuous wave laser. It promises the design of a new clock that would possibly have very interesting properties combined with a potentially quite simple setup. It would avoid complex ion traps and simultaneously addresses a very large number of nuclei. For this reason, the manuscript deserves publication in Nature. Nevertheless, I have a few suggestions that the authors should consider.

1) Linewidth: Obviously the most crucial next question if one really wants to build a reasonable accurate clock based on a 229Th doped crystal is how the linewidth can be reduced. This question should be addressed in more depth even when only speculations can be given at this stage. Questions that naturally arise is what are the factors that make the linewidth 9 orders of magnitude larger than the natural linewidth? Is it dominated by the magnetic dipole as the authors write? Is it true that it is „…ultimately limited by magnetic dipole interaction…“? Ultimate means this can never be surpassed. In that case I would probably be fair to say that there is no hope that the 229Th doped crystals will never be a good clock. Even then, in my view the work deserves publication in Nature but the reader should be supplied with an honest estimation of the prospects.

2) Stability: It would be useful to have a similar discussion for the stated stability of 2e-12 in 1sec averaging time. This is worse than a simple quartz. A discussion of how this limit might be circumvented in the future is particularly important as the main promise of the 229Th doped crystal is the large number of nuclei, i.e. a superior stability. The discussion so far is not sufficient. The suggested increase of the crystal length and the probe laser power is might do a little towards that goal, but it doesn’t convince for obtaining the missing several orders of magnitude. What are the prospects, even speculative to improve this to an technologically interesting regime.

3) Absorption measurement: The authors explain that one of the advantages of the absorption measurement is that it avoids the time lag due to the long lifetime of the excited state. This seems to be only true when the ground state is not depleted. „… the nuclear ground state population is not depleted significantly and therefore the absorbed VUV laser power is approximately time-independent.“. This important information is given on page 6 for the first time, as far as I can see, but is required to understand the statement in abstract already: “This eliminates the slow nuclear fluorescence decay from the detection process and offers a considerable advantage for clock operation through fast signal acquisition.“. Eventually the assumption of an undepleted ground state may break down when one finds a way to make use of the main feature of the 229Th doped crystal, that is, addressing a large number of nuclei at the same time. A discussion of the transient effects of the absorption signal on a high-performance clock would strengthen the motivation for this method, just like for the first two issues above.

4) Background signal: The main issue with absorption measurement is that the signal sits on a large and noisy background. This problem is not sufficiently discussed and also obscured to some extend in the way the data is presented. No value for the background I_0 is given and the signal in fig,3b is normalized. In this way the error bars lose their reference. One cannot simply take the square root to see how it compares to the shot noise. The latter is presumably dominated by the unrevealed background signal. Similarly, „the absorption probability is of 0.006*Gamma_eg/Gamma..“ without revealing what the symbol Gamma stands for and without giving a number.

5) Laser linewidth: „…a larger linewidth of ≈ 1 MHz, that exceeds the linewidth of the VUV laser.“ So what is the linewidth of the VUV laser? Along the same line: “We find spectra in which the laser linewidth is clearly below the nuclear transition linewidth to be best described by Lorentzian profiles.” Without any value, not even an estimate of the laser linewidth, one does not know what this statement exactly means.

6) Modelling: To model the line shape the authors “… assume a Cauchy-Lorentz distributed EFG…” Why that distribution? I would expect that valid statistical arguments are used to derive this distribution that Wikipedia describes as the “canonical example of a pathological distribution since both its expectation value and its variance are undefined.” The authors should explain in more detail why a distribution that doesn’t seem to be physical shows up here.

7) Line strengths: Inspecting the centers in fig.1, I would conclude that the number of D-centers are quadratically suppressed as it requires two Th atoms close by. However, this does not show up in the relative line strength of fig. 2b. Are the relative line strengths compensating for this? Again, the normalization may cover some of the important features. Have there been different normalizations applied to the different lines or is there a common factor that all signals have been divided by? This should be explained and presented more clearly.

In addition, I have a few suggestions concerning language and style that the authors might want to consider:
I find the 3D rendering in fig.3 difficult to read.

I cannot recognize the slit or anything that looks like a slit. Maybe a sketch is better than a 3D rendering. How does the light penetrate the MCP to reach the camera? The light from the collection mirror seems to a have kink in free space. The latter mirror collects *fluorescence* which is then converted into *phosphorescence* before it is detected. It would be helpful to repeat in here which of the centers in part b has the small quadrupole shift, maybe referring to fig.2.

„Exploiting the full potential of a Th-229 solid-state nuclear clock requires a laser source with a linewidth that is smaller than the nuclear linewidth“. What are the prospects of such a sub mHz laser?

„The optical transmission of the crystal at the nuclear transition wavelength was measured to be ≈ 40%.“ One may wonder here if the off resonance transmission is different form 40%.

„…vary more than the indicated error.“ I guess this is rather an uncertainly than an error.
A reference might be appreciated when the random quasi phase-matching is mentioned. This method is not yet widely known.

Referee #4

(Remarks to the Author)
I co-reviewed this manuscript with one of the reviewers who provided the listed reports.

Version 1:

Reviewer comments:

Referee #1

(Remarks to the Author)
Report on the revised manuscript of Nature ms 2026-04-10959A by Morawetz et al.

The authors have provided a revised version of their manuscript based upon the remarks, comments and suggestions by all three refereeing parties. In addition, a detailed response letter is attached covering all individual aspects and requests. We have thoroughly studied both the replies and explanations by the authors to our points as well as the revision and corrections which have been made. They rather completely address the recommendation of the referees, few other points can be accepted from our side, following the explanations of the authors. However, a few minor imperfections or misinterpretations have occurred and remain, for which we would like to request a second step of revision before final acceptance by Nature. These are listed in detail as referring to the revised version of the manuscript:

1. Page 1: the introduction of „crystal" in the last line will require correctly: " in a calcium fluoride crystal…" (add "a")

2. Page 3, 2nd paragraph: The sentence should more precisely read "…. changes in transmitted power in phase correlation with the modulation frequency (see…"

3. Page 3, 3rd paragraph: The repetition of the term „coherence time" should be omitted by writing "…is estimated to be below 10 ms, being significantly reduced due to…"

4. Figure1: This drawing is now much better and more easily understandable. At this point, we suggest to increase the size of the labelling text significantly for better readability – there is sufficient space available – and to clarify change "collection mirror" to "elliptical collection mirror" in consistency with the text body.

5. Page 5, 3rd paragraph: The repetition of „traceable to“ could be avoided – a suggestion would be "traceable to either an active H-maser locked to Coordinated…", if this is correct.

6. Page 6, 1st paragraph: In their response to our first report, the authors give a nice explanation about the process of the focusing of the light from the crystal (sitting in the first focus of the elliptical collection mirror) towards the slit (located at second focus of this mirror). Not all readers of Nature will be physicist, knowing about the imaging properties of elliptical mirrors – this nice explanation should be copied into the manuscript.

7. Fig. 2: The alterations of the drawing have somewhat contributed to clarity but still some further optimization of this important visualization of the data is recommended:
-Fig. 2a should have similar width like the graph of data of Fig 2c. This would lead to closer congruence of the lines indicated in panel a) and the signals shown in panels b) and c) It would then also allow to show explicitly the noise in the graph of the missing D-center 1/2 3/2 component in Fig. 2b, fluorescence, as has been requested by us in the report on the original version, and which we would like to see in the final version, especially also to aid non-specialist readers, who will expect one panel of fluorescence per panel of absorption. Panel d) could then still be drawn besides the other three panels, which will all take up the same amount of horizontal space, and will then no longer intrude into the other panels of the Fig., which is presently the case.
-The internal labelling of the O-center should include the word "unresolved" on an equal footing as the assignment of the spins of initial and final state in the D-center panels.

8. Fig. 4: we accept the authors request to keep this panel in the manuscript; the last sentence in the caption should be changed to "The (very narrow) quadrupole structure is not resolved." to be more precise.

Christoph Düllmann and Klaus Wendt

Referee #2

(Remarks to the Author)
The authors have provided comprehensive point-by-point responses to the reviewers' comments, supplemented and revised relevant content as required, and added explanatory notes. The revised manuscript features more rigorous presentation. The research findings reported in this paper represent a significant advance in the field of thorium-based solid-state nuclear clocks, and the revised manuscript is suitable for publication in this journal.

Referee #3

(Remarks to the Author)
The authors have included most of my suggestions. Some of which they didn't presumably because my intentions were not clear. I am trying again below. Reading the manuscript again, I noticed two more issues that could be solved to improve it.

- The manuscript still misses a discussion of how to achieve the prospects given in the first sentence, that is the promise of an "optical nuclear clock of very high accuracy and stability". All parameters are far away from that and one would expect at least some speculations how the many orders of magnitude could be gained in terms of linewidth, systematics and stability. Such a discussion would be helpful even if it was speculative at this time. "…we do not expect to jump over the frequency instability of high-finesse optical cavities at short times." Not even the best optical clocks can do that. However, they operate with typically 1e6 atoms while the potential number of addressable Th-229 could at the mol scale. Stability scales as sqrt(N). To me this is enough motivation to speculate.

- Fig1. is the MCP imaged on the CCD camera or just detected as the caption says? I do not recognize a lens for imaging.

- Line scan duration: "…crystal is periodically illuminated for 800 s, followed by a 800 s detection interval…"; "…scanned in 50 kHz steps over the ≈ 1 GHz rang…"; "Each transition is scanned twice…". Adding the numbers I conclude one line scan too 2yrs of uninterrupted operation. Later is says "The operation of an optical clock requires a signal to stabilize a laser to the reference frequency." Does that mean the servo bandwidth is limited to 1/2yrs? Please clarify how long the line scans really take.

- Again, shot noise:" The absorption and the error signals shown in Fig. 3 and Fig. 2c (absorption measurement of 6 lines) are not normalized." I think they are. This is what per cent = normalized to 100, literally means. "In this case the lock-in technique is often used to reduce an influence of a background…" The lock-in is a low pass filter that reduces the bandwidth. It does not remove the shot noise. The latter is proportional to the bandwidth Bw. In order to verify the magnitude of the error bars in fig.2b/c I would need the number of photons during 1/Bw. Because the signals normalized, this important piece of information is lost. At least I can not find it in the caption.

- Again: "the optical transmission…at the nuclear transition wavelength was measured to be \approx 40%". Technically correct but language wise very confusing if one does not mention that the absorption is also \approx 40% off resonance. Reading this I would falsely conclude that the absorption depth is \approx 40%. This statement comes before it is clear that this dip is actually less than 1%.

- Again, Cauchy: I have learned that the EFG shift is ~1/r^3 weighted with the probability ~r^2 this leads to a logarithmically diverging shift, just as with the Cauchy distribution. Therefore, the changes made to the manuscript do not solve resolve this issue, at least not for me, but even deepened it.

- Again, the suppression of the D-centers. I learned from the authors reply why the D-centers are not quadratically suppressed as everyone would expect. However, the information that it is due to clustering and that the lines are not normalized to different 100% levels is only given to me, not to the reader. At least I didn't find it in the caption.

Open Access This Peer Review File is licensed under a Creative Commons Attribution 4.0 International License, which permits use, sharing, adaptation, distribution and reproduction in any medium or format, as long as you give appropriate credit to the original author(s) and the source, provide a link to the Creative Commons license, and indicate if changes were made.
In cases where reviewers are anonymous, credit should be given to 'Anonymous Referee' and the source.
The images or other third party material in this Peer Review File are included in the article's Creative Commons license, unless indicated otherwise in a credit line to the material. If material is not included in the article's Creative Commons license and your intended use is not permitted by statutory regulation or exceeds the permitted use, you will need to obtain permission directly from the copyright holder.
To view a copy of this license, visit https://creativecommons.org/licenses/by/4.0/

Dear Editor,

We thank all four reviewers for their time and valuable feedback. We have revised the manuscript to address their comments and suggestions. Below, we provide a point-by-point response (reviewers' comments in red/black, our replies and manuscript changes in blue). We provide a 'colored' Version of the manuscript that highlights all changes made together with a plain version with all changes implemented.

Reviewer #1 (Remarks to the Authors):

Title: the title suggests a cw laser to be a necessary prerequisite for the presented work, and it also suggests that this is a new achievement. In its current form, the title even starts with "cw nuclear laser", which is misleading. For us, the essence of the work is not the continuous-wave laser (which was already published by the group in their ref. [5], and where another group operates a stronger cw laser, see ref. [11] in the present manuscript) but establishing absorption spectroscopy to allow for substantially shorter cycling times in state-interrogation. The key is in the narrow linewidth, which requires a cw laser. A suggestion for the title is "Narrow bandwidth laser absorption spectroscopy in the nuclear isomer transition of thorium-229".

Reply.

We agree the current title is hard to understand/interpret, hence for more clearity we rephrase the title to "Continuous-wave laser absorption spectroscopy of the Th-229 nucleus". However, we refrain to use term narrow bandwidth as this is not an exclusive property of CW lasers. Femtosecond laser with coherent pulses (e.g. fs combs) can also achieve linewidth as narrow as CW laser in single modes of the emerging frequency comb. The phrase/term "CW laser" in title highlights the ease and possibility with which some of the well established spectroscopy and laser stabilization techniques/methods can be performed for further development of nuclear clocks.

Abstract: in line with the above, the statement on line 7 on "continuous wave" should state "narrow bandwidth", i.e., "can be excited with a continuous-wave narrow bandwidth laser source". The abstract closes with the statement "..., promising nuclear resonance lines that are less sensitive to the lattice spacing". In a broad journal like Nature it might be appropriate to state explicitly why this is advantageous. Also, the question of "less sensitive than what" lingers. One could replace "less sensitive" by "quite independent" in this last sentence.

Reply.

We agree with the referee on to the first suggestion. The expression "less sensitive" stands in direct relation to the preceding sentence on earlier observed Th-centers. We prefer to keep the statement like this.

Body text:

Page 2: The natural linewidth is first given as being in the millihertz range, and then as being crystal-field-broadened to 10-100 kHz. The authors then write that exploiting the full potential of a Th-229 solid-state nuclear clock requires a laser source with a linewidth that is smaller than the nuclear linewidth, but it is left open which of the two values stated earlier this refers to. It follows implicitly from the text on page 3, but it would be helpful at this point if this were stated more explicitly. Also, as far as the quoted millihertz linewidth goes, it should be added that this is theoretically predicted (including a reference) but not experimentally determined to date. In addition, we consider "smaller than the nuclear linewidth" too strict a requirement; "the same order" would appear more fitting to us. Finally, a reference should also be added for the 10-100 kHz crystal-field-broadened linewidth.
Reply.
To avoid a confusion of readers, we cut out the sentence about the millihertz linewidth in ions, because this is not the subject of the present work. We added the reference which gives the information about the crystal-field-broadened line and rephrased the requirements to the laser linewidth.

Page 2: For the non-expert reader it might be helpful to add a side remark of why the requirement of a narrow linewidth is only fulfilled in continuous wave lasers, but not in pulsed ones. Fundamentally, the narrow linewidth is the physics requirement.
Reply.
The narrow linewidth can be obtained either with a CW laser or with a pulsed laser that maintains coherence between pulses, e.g. a femtosecond frequency comb. However, the additional non-resonant comb modes that are unavoidably present in radiation spectra, result in increased crystal disturbances such as light-induced quenching (see e.g. Schaden, F. et al. Phys. Rev. Res. 7, L022036 (2025).) and a reduction in the signal-to-noise in the transmission signal. We added an explanatory remark to the manuscript.

Page 2: "in the VUV and possess" $\rightarrow$ "in the VUV spectral region and possess"
Reply.
Thank you for this comment, we changed the text accordingly.

Page 3: the first text line states that the laser linewidth can be narrowed, but there is no quantitative statement to which linewidth this is possible. This should be added.
Reply.
To avoid a reduction of the absorption signal, the laser linewidth should be upper bounded by the nuclear transition linewidth under investigation. We rephrased this sentence in the manuscript.

Page 3: the sentence “A spectroscopic signal can be recorded by modulating the laser frequency over a fraction of the absorption linewidth…” likely refers to Pound-Drever-Hall-locking, which should be added.
Reply.
We do not refer to a Pound-Drever-Hall lock which generates an error signal obtained in reflection as an interference of a cavity signal with high frequency modulation sidebands which are not passing through the cavity resonance. The method used here is similar to a standard lock-in technique used for locking to resonances in absorption vapor cells. We added relevant citations to the main text.

Page 3: “The ability to perform absorption detection is pivotal for the operation of a solid-state nuclear clock. It probes…”: does the “It” starting the next sentence refer to the absorption detection or to the operation of a clock”?
Reply.
We agree with the referee, we changed "it" to "the absorption detection".

Page 3: the authors should add a sentence explaining why the coherence time in the crystal is so short. Is it the influence by the lattice, and is there a citation available for this?
Reply.
We understand the sentence is unclear and we adapted it for clarity.

Page 3: “It minimizes the detrimental effects on the signal from non-resonant VUV photons. These may…”: These → Effects. Otherwise the “these” refers to the photons.
Reply.
We agree, so we changed the text accordingly.

Page 4: How was the optical transmission of the crystal at the nuclear transition wavelength measured to be 40%? By the absorption spectroscopy presented in the paper? What is the precision of this value?
Reply.
The transmission of the crystal at the nuclear transition was measured to be 40(5)%, before the experiments, using a VUV monochromator in a broad spectral range. Measurements of the transmission during operation are often unreliable as they are very sensitive to the alignment. Additionally, there can be a large difference between surface and bulk transmission of Th:CaF2, which has been extensively described in Beeks et al. PRB (Ref. 21) and Beeks, K. Ph.D. thesis, Technische Universität Wien (2022) (Ref. 22).

Page 4: Fig. 1: (1) this figure is almost impossible to comprehend for non-specialists, not least as absorption and fluorescence spectroscopy are not clearly separated. Also, the crystal, which is at the center of the experiment, is barely visible; (2) the component deflecting the green phosphorescence is not labeled; (3) the slit is drawn in a manner as if this and the MCP formed a single part.

The figure is ideally redrawn with a clearer separation of all components; (4) the role of the slit is not mentioned in the caption; (5) the fluorescence appears to be drawn including unphysical angles; it is not evident that the mirror emits the phosphorescence that is eventually detected by the camera; (6) in b/w printouts, the O-center and D-center zoom regions appear exactly identical. The authors should select colors with different gray tones for Ca and Th-229; (7) why is there a central Th atom indicated in the center of the cell of the D center inset? (8) there should be consistence in the abbreviations in the figure and in the caption.
Reply.
Thank you for this comment, we agree that the 3D schematic in fig. 1 might be difficult to comprehend. Therefore we have replaced it with a 2D representation of the setup which should sufficiently simplify the figure (1-5). We have also adapted the colors of the zoom-in regions to avoid confusions in b/w printouts (6). The D-center refers to a Th dimer configuration, meaning two Th atoms are replacing two Ca atoms in the lattice as nearest neighbors as represented in the zoom-in (7). Lastly, we have adapted the abbreviations for consistency (8).

Page 5: the top part of the page describes a first laser generating 1187 nm light, which is frequency quadrupled to 296.8 nm, after which frequency doubling to 148.4 nm is done. Later on, separation of the desired 148.4 nm light from "fundamental UV" radiation is performed. However, "fundamental" is the light at 1187 nm, not at 296.8 nm.
Reply.
We agree with the comment. To avoid confusion we have rephrased these statements in the manuscript and added a statement about the dichroic mirrors for the separation of 149.4 nm and 296.8 nm in the methods.

Page 5: it would be useful to discuss the width of the fundamental linewidth and the increase in width due to the different doubling mechanisms.
Reply.
Unfortunately, it is quite complex to give a fully correct answer for the linewidth broadening over 3 SHG stages. For the narrow linewidth operation the IR laser is locked to the high-finesse cavity and has the instability of 10^{-15} at 1 s. Assuming the fundamental laser linewidth is broadened only by white frequency noise, the VUV linewidth at short times (0.1-1 s) should be <100 Hz (an enhancement/broadening by factor of 64 in VUV). An additional broadening appears from the upconversion of phase noise in our system. The phase noise depends also on the laser and tapered amplifier operation regime, and a lockbox bandwidth/-gain adjustment. The influence of these effects is difficult to study with only one laser system available. We provided some measurements with scanning cavities at 300 and 600 nm which were not precise but somehow agree with our estimations. The estimations give $\sim$ 1 kHz VUV linewidth for the conversion of the cavity stabilized laser system. While we can't directly measure the linewidth, we give the estimations based on the resonance linewidth. Therefore, we give

the value of $\leq$ 10 kHz in the main text. The same approach is also used in Ooi, T. et al. Nature 650, 72–78 (2026), where 1 kHz linewidth was estimated for the VUV optical comb based on the Th spectral lines. In this work the crystal with lower concentration was used resulting narrower Th resonances. We extended the discussion of the VUV laser linewidth in the main text and in Methods.

Page 5: define UTC(BEV)
Reply.
We changed the relevant parts to "traceable to either Coordinated Universal Time (UTC) or a Yb+ single ion clock." and "...de-drifted with a feedback loop to an active H-maser traceable to Coordinated Universal Time (UTC) or to a Yb+ single ion clock." to clarify the used references.

Page 5: is the "elliptical mirror" described in the text identical to the "collection mirror" shown in Fig. 1? If so, identical nomenclature should be used.
Reply.
For consistency in nomenclature we have rephrased it as 'elliptical collection mirror' to highlight that this mirror has two foci, one where the crystal is placed and the other one where the slit is placed.

Page 6: The authors state that "the frequency is stepwise shifted by 2 MHz" in the absorption measurements. In connection with Fig. 2, however, where the text states that the Fig. shows fluorescence and absorption measurements, there are spectra shown with much smaller step size, tracing the linewidths on the level of less than 100 kHz. So other steps were used as well. The authors should also write explicitly to panel b of Fig. 2 that this shows fluorescence measurements. It can be inferred from the sign of the signal, and in panel c it can also be inferred from the y-axis label that these are the absorption measurements, but explicit statements are helpful especially for non-specialists. It would also be interesting to learn what the linewidths are, which seem surprisingly similar in both spectroscopy methods.
Reply.
The 2 MHz is the amplitude of the frequency modulation that is used in the differential absorption detection. It is not related to the step size of the spectra. To clarify we change "A stepwise frequency shift with an amplitude of 2 MHz..." to "is the detected intensity 2 MHz detuned off-resonance... alternating between the two frequencies..."

For the wide scan the width of the absorption resonances is given in Tab.1 and is limited by laser linewidth (see our explanation about the laser linewidth in the main text). The fluorescence spectral width is in agreement with the absorption lines, as the underlying nuclear transition width and laser linewidth are comparable. The y-axis label in 2b is changed from 'normalized count rate' to 'normalized fluorescence'.

Page 6: the expression $\sqrt{1/15} - \sqrt{2/3}$ reads as a mathematical expression,

i.e., a subtraction, but it means a range, i.e. $\sqrt{1/15} - \sqrt{2/3}$ -"... the estimate of the absorption probability is of 0.006 Γ_{eg}/Γ..." → is of the order of?
Reply.
To avoid confusion between subtraction and range we have rephrased the statement as 'ranging between $\sqrt{1/15}$ and $\sqrt{2/3}$'
We adapted the following sentence: "the estimate of absorption probability ranges between 0.04% and 0.4% depending on the specific transition.", for more clarity. Also, we corrected a typo in the expression for the absorption probability above: $\rho l \lambda^2 \Gamma_{eg}/(6\pi\Delta\omega)$ instead of $\rho l \lambda^2 \Gamma_{eg}/(6\pi\Delta\nu)$.

Page 6: after "In absorption spectroscopy, we observe six lines" the authors could add "in contrast to fluorescence spectroscopy where only five are observed.
Reply.
We think that such a statement would put too much emphasis on the difference. It is only a question of signal to noise ratio, therefore we keep it as it is.

Page 7: the authors note that some of their reported line centers deviate from literature by more than 3 sigma, and give a tentative explanation. This suggests that similar fluctuations will occur in future studies using different crystals. What are the implications of this fact for stable clock operation?
Reply.
At the moment we cannot expand further than our current tentative explanation of the observed line center deviations being caused by local doping inhomogeneities or local stress. The effect of these fluctuations will be subject of further investigations, as we agree that this might significantly impact accurate clock operation.

Page 7: what is the minimum laser linewidth the authors have achieved?
Reply.
We partially answered this question in the reply to the question on Page 5. In the text we give only the upper bound of the laser linewidth. The nuclear transition width observed in our experiment agrees with the experiment of Ooi, T. et al. Nature 650, 72–78 (2026), where 1 kHz linewidth was estimated for the VUV optical comb based on the Th resonance width. We can only confirm that the final VUV linewidth is significantly narrower than the resonance linewidth and lies in the range of ≤ 10 kHz.

Page 8: in Fig. 2 a, the panel should be divided in vertical direction into an upper panel for the O center and a lower panel for the D center. In the current form it is very hard to understand.
Reply.
To keep the Figure compact we prefer to keep the O-center and D-center in the same panel. This also reflects how the line spectrum is observed in the experiment prior to our assignment of the lines to different doping configurations.

Page 8: the center of the quadrupole-structure of the D center is once given

as “the black line” in panel Fig. 2d and as “the black dotted line” in Fig. 2a. Ideally, the same is used in both panels, and in the text, and ideally as solid line.
Reply.
We agree and have adapted fig. 2 accordingly.

Page 8: in between Fig. 2b and Fig. 2c, the nomenclature “$3/2 \rightarrow 1/2$” on the one hand and “O-center” on the other hand is used. We suggest to add “D-center $3/2 \rightarrow 1/2$” etc. space permitting. Or, even better, the panels of the O center should be separated from the panels of the D center.
Reply.
We have added “D-center" to the nomenclature, as suggested.

Page 8: the arrangement of the frequency labels on top of Fig. 2a is not helpful. It leads to an inferred correspondence of the frequencies of panel a with panels b and c, which is not the case. The labels should be given below panel a to avoid this.
Reply.
We agree and have moved the frequency labels to the bottom.

Page 9: In the second sentence in the Discussion part, the authors refer to “This line”, but it is not explicitly stated which one this is. The authors then express their conjecture on the presence vs. absence of said line, but the reader cannot get any information on the implications on this. A sentence or two on this would be helpful to qualify the relevance of this discrepancy among different studies.
Reply.
For clarity, the statement is rephrased to ‘The O-center line...’. We further clarified the sentence regarding quenching on page 9.

Page 10: Fig. 4 is not necessary and can be omitted.
Reply.
We disagree with this statement. Fig. 4 shows a O-line detected with narrow laser linewidth and therefore, with better resolution than that in Fig. 2. It demonstrates that the internal quadrupole structure of this line is not resolved even with narrower laser linewidth. In addition, we also emphasize it in the figure capture.

Reviewer #2 (Remarks to the Author):

Comment (1) The manuscript assigns the O-center to an O_h high-symmetry configuration. Theoretically, the electrostatic field gradient at the nuclear site under this symmetry is spherically symmetric, and the EFG asymmetry parameter η should be strictly zero. However, when fitting the EFG of the O-center, the paper fixes η to 0.57 (the value of the D-center) and only states that η has no substantive effect on the results. This assumption directly contradicts the high-symmetry property of the O-center, which may introduce systematic bias in the estimation of the upper bound of V_{zz} and undermine the core conclusion of the extremely low EFG of the O-center

Reply.

The asymmetry parameter is defined as $\eta = (V_{xx} - V_{yy})/V_{zz}$, where V_{zz} is the largest absolute component of the diagonalized EFG tensor. Under O_h symmetry, V_{zz} vanishes and η is consequently undefined. Our goal is to derive an upper bound on V_{zz} from the experimental data. Any deviation from ideal O_h symmetry breaks the local symmetry, reorients the EFG principal axes, and leads to nonzero V_{zz}. Consequently, no symmetry constraint on η remains, and any value from 0 to 1 is in principle accessible. Since we lack a microscopic model of the EFG distribution, we adopted $\eta = 0.57$ (the D-center value) as a simplifying assumption.

To assess the sensitivity of our conclusions to this choice, we repeated the fit, including η fixed to 0 and 1. The fitted values are $V_{zz} = 0.033 \pm 0.027$, and $0.006 \pm 0.031\,\mathrm{V/Å^2}$ for $\eta = 0.00$, and 1.00, respectively. In all three cases, the upper bound on V_{zz} is $0.1\,\mathrm{V/Å^2}$, confirming that the choice of η does not affect this bound.

Comment (2) The observed absorption signal amplitude is only 0.02%–0.36%, which is an extremely weak signal. However, no control experiments have been conducted to exclude non-nuclear transition contributions. It is impossible to completely rule out interference from non-nuclear factors such as crystal color centers, impurity absorption, laser-induced transient defects, and background fluctuations of Cherenkov radiation.

Reply.

We would not describe the observed absorption signal as extremely weak: It could be detected with $> 5\sigma$ confidence within a few seconds. Even without specific control experiments, we regard the evidence that what we observe is nuclear spectroscopy as compelling. We would not expect any of the potentially non-nuclear factors mentioned by the referees to produce a spectrally narrow (< 100 kHz) resonance feature. We observe the clear correlation between absorption and fluorescence resonances, the consistency of the resonance width and relative amplitudes that match the expected absorption value, within this study and with the results from the earlier experiments (especially Refs. 1 and 3), where control experiments have been performed. The recorded amount of Cherenkov radiation is strongly reduced for the fluorescence path by spectral filtering with the grating and a very small solid angle in case of the transmis-

sion measurements. In both cases, this background is small compared to the recorded signal. In addition, no relevant laser-induced transient defects have been found in earlier studies for the here applied power < 1 nW.

Comment (3) The manuscript only provides an estimated fractional frequency instability of 2×10^{-12} at 1 s averaging time based on the shot-noise limit, but lacks quantitative noise analysis and long-term stability data. It is impossible to determine the current noise-limiting factor (shot noise / technical noise) of the system, nor to evaluate the reliability of absorption detection in the long-term operation of nuclear clocks.
Reply.
It is true that in this work we focus on the spectral analysis and the short-term signal-to-noise ratio which is consistent with shot-noise in the photon detection. The stated frequency instability for clock operation is only meant as an indication of what could be possible given the present experimental parameters. A more detailed analysis of long-term stability is beyond the scope of this paper.

Comment (4) All experiments were performed at a single temperature of 294.7(5) K, and data on the dependence of nuclear transition frequency, linewidth, and absorption amplitude on temperature are missing. The temperature coefficient is the most important source of systematic error for solid-state nuclear clocks and directly determines the required temperature control precision of the clock. It is recommended to perform a systematic characterization of the effect of temperature on the spectral and frequency performance of the crystals.
Reply.
The temperature dependence for segments from the same parent crystal have already been reported in Ooi, T. et al. Nature 650, 72–78 (2026). Our present apparatus did not permit a significant deviation from room temperature. The primary topic of this work is the demonstration of the absorption spectroscopic techniques and methods with a continuous-wave laser. A detailed analysis of systematic uncertainty contributions is beyond the scope of this work.

Comment (5) All experiments were performed on a single crystal with a bulk 229Th concentration of 6.6×10^{15} mm^{-3}, and no quantitative correlation data between concentration and the O/D center ratio, linewidth, absorption amplitude, and signal-to-noise ratio are provided. It is impossible to determine the optimal doping concentration for nuclear clocks, nor to verify whether reducing the concentration can narrow the linewidth of the O-center and resolve its quadrupole structure. The authors are suggested to supplement the study on the effect of 229Th doping concentration on crystal performance.
Reply.
The O/D center ratios for crystals of different doping densities, including a cut of X2, has been previously studied in T. Hiraki, et al. arXiv:2509.00041. Further, the dependence of the linewidth on doping concentrations, including a cut of X2, has been investigated in Ooi, T. et al. Nature 650, 72–78 (2026). We agree that the question about the optimal doping density is interesting. For this

reason we discuss it in L. Toscani De Col, et al. arXiv:2606.04997.

Comment (6) The theoretical isomer shift (3.526 MHz) is in good agreement with the experimental value (3.991±0.013 MHz), but the calculation relies on the literature value $\Delta\langle \mathrm{R}^2\rangle$=0.0105(13) fm^2, and its 12% relative uncertainty has not been quantified. In addition, independent verification methods for the electron density difference using other experimental techniques are not discussed. The formation conditions of the O-center and the coexistence mechanism with thorium clusters also remain unclear.

Reply.

We acknowledge that error propagation was absent in the submitted manuscript. In the revised version, we have performed a systematic propagation of the uncertainty in $\Delta\langle R^2\rangle$ to the final isomer shift. Regarding verification of the electron density at the nucleus, we note that the recently measured temperature dependence of the isomeric shift [Higgins et al., PRL 134 (2025)] would provide a consistency check on $\rho(0)$ in the CaF_2 host. However, accurate simulations of such temperature effects require detailed treatment of lattice expansion, anharmonic contributions, and concentration-dependent mechanisms. We therefore prefer to defer this analysis to a dedicated study. Regarding formation conditions of the O-center and coexistence with thorium clusters, we note that recent work [Hiraki et al., arXiv:2509.00041] reports a lower relative occurrence of the D-center at lower doping concentrations, with the D-center fraction increasing at higher concentrations. This trend is consistent with a picture in which kinetic processes during crystal growth govern the defect distribution: at low Th concentrations, isolated Th (O-center) is statistically favored, while at higher concentrations, the increased probability of two Th atoms in close proximity leads to a larger number of dimer (D-center) formation. A quantitative understanding of these kinetics lies beyond the scope of the present study, and we believe that a comprehensive discussion of crystal growth conditions would distract from the paper's central spectroscopic findings. Thus, we added a brief remark of this hypothesis to the methods section of the revised manuscript.

Comment (7) It is well known that thorium clusters (such as the D-center) are common in Th:CaF_2, but the manuscript does not clarify the stable formation conditions and charge compensation mechanism of the O-center.

Reply.

Th^{4+} substituting for Ca^{2+} creates a +2 charge imbalance. To preserve the O-center's high symmetry, we propose that charge compensation occurs via nonlocal defects, such as calcium vacancies or fluorine interstitials at distant lattice sites [Hiraki et al., arXiv:2509.00041]. As stated previously, the formation of O-center and D-center configurations may originate from kinetic processes during crystal growth, and a detailed microscopic understanding of these formation mechanisms lies outside the scope of the present work.

Reviewer #3 (Remarks to the Author):

1) Linewidth: Obviously the most crucial next question if one really wants to build a reasonable accurate clock based on a 229Th doped crystal is how the linewidth can be reduced. This question should be addressed in more depth even when only speculations can be given at this stage. Questions that naturally arise is what are the factors that make the linewidth 9 orders of magnitude larger than the natural linewidth? Is it dominated by the magnetic dipole as the authors write? Is it true that it is „. . . ultimately limited by magnetic dipole interaction. . . "? Ultimate means this can never be surpassed. In that case I would probably be fair to say that there is no hope that the 229Th doped crystals will never be a good clock. Even then, in my view the work deserves publication in Nature but the reader should be supplied with an honest estimation of the prospects.
Reply.
The magnetic interaction is suspected to broaden the lines of the isomer transition to 100-1000 Hz. We suspect that the dominant broadening mechanism in our crystals is trough (inhomogeneous) strain, leading to the observed linewidth on the order of 100 kHz. We expect that this strain could be circumvented in crystals in which Thorium is part of the stoichiometric crystal structure, such as ThF4. Further Solids containing only isotopes without nuclear spin have been proposed (H. Morgan, et al. arXiv:2503.11374) which might enable sub 100 Hz linewidth.
For the accuracy of a solid state clock, we think that not the linewidth by itself, but systematic shifts induced by strains in the crystal are likely the limiting factor. These might again be reduced in crystals in which Thorium is part of the stoichiometric crystal structure.
We think that discussing clock performances based on other host materials in more detail, is beyond the scope of this paper.

2) Stability: It would be useful to have a similar discussion for the stated stability of 2e-12 in 1sec averaging time. This is worse than a simple quartz. A discussion of how this limit might be circumvented in the future is particularly important as the main promise of the 229Th doped crystal is the large number of nuclei, i.e. a superior stability. The discussion so far is not sufficient. The suggested increase of the crystal length and the probe laser power is might do a little towards that goal, but it doesn't convince for obtaining the missing several orders of magnitude. What are the prospects, even speculative to improve this to an technologically interesting regime.
Reply.
We modified/rephrased the concerning sentence now to make it more clear that the shot noise is a trend that can be expected to extend beyond the indicated time of 1 s. We also added a few sentences for better explanation and prospects of nuclear clocks performance. Using a nuclear resonance as a reference for optical clocks we do not expect to jump over the frequency instability of high-finesse optical cavities at short times. The major potential of Th solid state clocks can

be realized at long integration times (long term stability), where a high-finesse cavity instability starts to degrade. It is similar to feedback provided in modern frequency standards based on resonances in ions or neutral atoms.

3) Absorption measurement: The authors explain that one of the advantages of the absorption measurement is that it avoids the time lag due to the long lifetime of the excited state. This seems to be only true when the ground state is not depleted. „. . . the nuclear ground state population is not depleted significantly and therefore the absorbed VUV laser power is approximately time-independent.". This important information is given on page 6 for the first time, as far as I can see, but is required to understand the statement in abstract already: "This eliminates the slow nuclear fluorescence decay from the detection process and offers a considerable advantage for clock operation through fast signal acquisition.". Eventually the assumption of an undepleted ground state may break down when one finds a way to make use of the main feature of the 229Th doped crystal, that is, addressing a large number of nuclei at the same time. A discussion of the transient effects of the absorption signal on a high-performance clock would strengthen the motivation for this method, just like for the first two issues above.

Reply.

We now modified the concerned paragraphs on p.3 and p.6 to clarify that only a very small fraction of the atoms get excited. All our measurements were performed in the low saturation regime.

4) Background signal: The main issue with absorption measurement is that the signal sits on a large and noisy background. This problem is not sufficiently discussed and also obscured to some extend in the way the data is presented. No value for the background I_0 is given and the signal in fig,3b is normalized. In this way the error bars lose their reference. One cannot simply take the square root to see how it compares to the shot noise. The latter is presumably dominated by the unrevealed background signal. Similarly, „the absorption probability is of $0.006\Gamma_{eg}/\Gamma$.." without revealing what the symbol Γ stands for and without giving a number.

Reply.

We agree, that the absorption signal sits on the large and noisy background. It is quite similar to the signals in absorption cells, used for the frequency stabilization of lasers (like CH_4, I_2, and Rb stabilized lasers.). In this case the lock-in technique is often used to reduce an influence of a background low frequency noise. Here we use a similar method. The absorption and the error signals shown in Fig. 3 and Fig. 2c (absorption measurement of 6 lines) are not normalized. The absorption signal is given in the percentage of the absorption and the error signal in the corresponding slope units (given the Fig. 3 caption). The number of incident VUV photons (VUV power) on the PMT is also given in the main text on Page 5. The PMT quantum efficiency of 10% we added in the description of the experimental setup. The I_0 (photon flux detected on PMT) and the calculated shot noise limit are given in the Discussion.

Furthermore, we adapted the following sentence: "the estimate of absorption probability ranges between 0.04% and 0.4% depending on the specific transition.", for more clarity.

5) Laser linewidth: „…a larger linewidth of ≈ 1 MHz, that exceeds the linewidth of the VUV laser." So what is the linewidth of the VUV laser? Along the same line: "We find spectra in which the laser linewidth is clearly below the nuclear transition linewidth to be best described by Lorentzian profiles." Without any value, not even an estimate of the laser linewidth, one does not know what this statement exactly means.

Reply.

To avoid the confusion, we erased the part of the sentence: that exceeds the linewidth of the VUV laser. This clear statement is not required to be emphasized in the text. We extended the discussion about the VUV linewidth in the main text and in Methods.

6) Modelling: To model the line shape the authors "… assume a Cauchy-Lorentz distributed EFG…" Why that distribution? I would expect that valid statistical arguments are used to derive this distribution that Wikipedia describes as the "canonical example of a pathological distribution since both its expectation value and its variance are undefined." The authors should explain in more detail why a distribution that doesn't seem to be physical shows up here.

Reply.

We thank the referee for raising this important point. We model point defects, whether thorium or otherwise, as independently and uniformly distributed throughout the crystal at low concentration. Each defect contributes to the EFG at the laser-excited thorium site as $V_{zz} \propto r^{-3}$. The probability of finding a defect in a spherical shell of radius r centered on the thorium nucleus is $p(r)dr \propto r^2 dr$. Changing variables from r to V_{zz} yields $p(V_{zz})dV_{zz} \propto V_{zz}^{-2} dV_{zz}$. The Cauchy-Lorentz distribution reproduces the correct behavior at its tails, in addition to being symmetric about the origin.

Regarding the undefined expectation value and variance: these are a consequence of the idealized model of a continuous, infinite crystal with no minimum defect-thorium separation. In a real crystal, physical constraints mitigate the problem: the crystal is finite, discrete, and a minimum defect-thorium distance imposes an upper bound on $|V_{zz}|$.

Since the precise microscopic origins of inhomogeneous broadening are not understood, the Cauchy-Lorentz distribution serves as a physically motivated model to enable meaningful comparison of linewidth parameters between O- and D-centers without requiring complete knowledge of the underlying mechanisms. We have added this justification to the revised manuscript.

7) Line strengths: Inspecting the centers in fig.1, I would conclude that the number of D-centers are quadratically suppressed as it requires two Th atoms close by. However, this does not show up in the relative line strength of fig. 2b.

Are the relative line strengths compensating for this? Again, the normalization may cover some of the important features. Have there been different normalizations applied to the different lines or is there a common factor that all signals have been divided by? This should be explained and presented more clearly.
Reply.
To answer the normalization question: All lines were normalized by the exact same factor. Secondly, although intuitive, it is not correct to assume D-centers are quadratically suppressed as it requires two Th atoms. The formation energies of these type of defects depend heavily on the specific atom, see Ma, Fengkai, et al. "The defect aggregation of RE3+ (RE= Y, La Lu) in MF2 (M= Ca, Sr, Ba) fluorites." Materials Research Bulletin 125 (2020): 110788. As the formation energies are different, the growth method then directly determines the population ratios, at which temperature did the crystal crystallize and how quickly was it cooled down? Then finally, aside from pure populations of defects, the O-center and D-center have different quenching strengths which will affect the amount of signal observed, see Hiraki, Takahiro, et al. "Laser Mössbauer spectroscopy of ^{229} Th." arXiv preprint arXiv:2509.00041 (2025). So making any direct conclusions on relative line strengths is likely an involved process which is beyond the presented work.

Language and style: I find the 3D rendering in fig. 3 difficult to read.
Reply.
We assume, the question refers to Fig. 1 (because there is no 3D in Fig. 3). For better readability we have changed the 3D drawing to 2D in the main text.

Language and style: I cannot recognize the slit or anything that looks like a slit. Maybe a sketch is better than a 3D rendering. How does the light penetrate the MCP to reach the camera? The light from the collection mirror seems to a have kink in free space. The latter mirror collects *fluorescence* which is then converted into *phosphorescence* before it is detected. It would be helpful to repeat in here which of the centers in part b has the small quadrupole shift, maybe referring to fig.2.
Reply.
We agree that the 3D setup schematic might be confusing, so we have replaced it with a 2D sketch.

Language and style: „Exploiting the full potential of a Th-229 solid-state nuclear clock requires a laser source with a linewidth that is smaller than the nuclear linewidth“. What are the prospects of such a sub mHz laser?
Reply.
Thank you for the remark. We clarified in the text that we are referring to the crystal field broadened linewidth and not the fundamental linewidth of the nuclear transition.

Language and style: „The optical transmission of the crystal at the nuclear transition wavelength was measured to be $\approx 40\%$.“ One may wonder here if the

off resonance transmission is different form 40%.
Reply.
The transmission of the crystal was measured using a VUV monochromator in a broad range. See citations: Beeks, K. Ph.D. thesis, Technische Universität Wien (2022) and Beeks, Kjeld, et al. "Optical transmission enhancement of ionic crystals via superionic fluoride transfer: growing VUV-transparent radioactive crystals." Physical Review B 109.9 (2024): 094111. Respective citations of these works have been added in the text/manuscript.

Language and style: „. . . vary more than the indicated error.“ I guess this is rather an uncertainly than an error.
Reply.
We agree and corrected it in the main text.

Language and style: A reference might be appreciated when the random quasi phase-matching is mentioned. This method is not yet widely known.
Reply.
We agree with this statement and added two citations which explain RQPM on page 5: Trabs, et al. Opt. Lett. 41, 618–621 (2016) and M. Baudrier-Raybaut, et al., Nature 432, 374 (2004).

On behalf of all authors,

Thorsten Schumm, Ekkehard Peik

Dear Editor,

We thank all referees for once more investing their time and continue to further improve the quality of the manuscript. We have revised the manuscript to address their comments and suggestions. Below, we provide a point-by-point response (reviewers' comments in red/black, our replies and manuscript changes in blue).

Also, we have slightly modified the abstract to comply with the word limit, as requested by the editor. Upon resubmission, we provide a 'colored' Version of the manuscript that highlights all changes made, together with a plain version, with all changes implemented.

On behalf of all authors,

Thorsten Schumm, Ekkehard Peik

Referee #1 (Remarks to the Author):

The authors have provided a revised version of their manuscript based upon the remarks, comments and suggestions by all three refereeing parties. In addition, a detailed response letter is attached covering all individual aspects and requests. We have thoroughly studied both the replies and explanations by the authors to our points as well as the revision and corrections which have been made. They rather completely address the recommendation of the referees, few other points can be accepted from our side, following the explanations of the authors. However, a few minor imperfections or misinterpretations have occurred and remain, for which we would like to request a second step of revision before final acceptance by Nature. These are listed in detail as referring to the revised version of the manuscript:

1. Page 1: the introduction of „crystal" in the last line will require correctly: " in a calcium fluoride crystal..." (add "a")

Reply:
Thank you for spotting this, we have added the missing 'a'.

2. Page 3, 2nd paragraph: The sentence should more precisely read ".... changes in transmitted power in phase correlation with the modulation frequency (see..."

Reply:
We have adapted the sentence in the main text.

3. Page 3, 3rd paragraph: The repetition of the term „coherence time" should be omitted by writing "...is estimated to be below 10 ms, being significantly reduced due to..."

Reply:
Thank you for this suggestion, it sounds better and we have changed the text accordingly.

4. Figure 1: This drawing is now much better and more easily understandable. At this point, we suggest to increase the size of the labelling text significantly for better readability – there is sufficient space available – and to clarify change "collection mirror" to "elliptical collection mirror" in consistency with the text body.

Reply:
The size of the labeling text is chosen to comply with nature rules for figures. We have changed the label to "elliptical collection mirror".

5. Page 5, 3rd paragraph: The repetition of „traceable to" could be avoided – a suggestion would be "traceable to either an active H-maser locked to Coordinated...", if this is correct.

Reply:
We agree with the referee and have improved the wording to "Measurements of the absolute VUV laser frequency are performed by referencing to a signal from an active H-maser traceable to Coordinated Universal Time (UTC) or an Yb+ single ion clock [26] established at the Austrian Federal Office of Metrology and Surveying (BEV) depending on the availability. The reference signal is delivered to the ATI via a Doppler-compensated fiber link."

6. Page 6, 1st paragraph: In their response to our first report, the authors give a nice explanation about the process of the focusing of the light from the crystal (sitting in the first focus of the elliptical collection mirror) towards the slit (located at second focus of this mirror). Not all readers of Nature will be physicist, knowing about the imaging properties of elliptical mirrors – this nice explanation should be copied into the manuscript.

Reply.

Thank you, we have added this explanation to the main text.

7. Fig. 2: The alterations of the drawing have somewhat contributed to clarity but still some further optimization of this important visualization of the data is recommended:

-Fig. 2a should have similar width like the graph of data of Fig 2c. This would lead to closer congruence of the lines indicated in panel a) and the signals shown in panels b) and c) It would then also allow to show explicitly the noise in the graph of the missing D-center $1/2 \rightarrow 3/2$ component in Fig. 2b, fluorescence, as has been requested by us in the report on the original version, and which we would like to see in the final version, especially also to aid non-specialist readers, who will expect one panel of fluorescence per panel of absorption. Panel d) could then still be drawn besides the other three panels, which will all take up the same amount of horizontal space, and will then no longer intrude into the other panels of the Fig., which is presently the case.

-The internal labelling of the O-center should include the word "unresolved" on an equal footing as the assignment of the spins of initial and final state in the D-center panels.

Reply.
As much as we would like to comply with the reviewers request: the full fluorescence profile on the $1/2 \rightarrow 3/2$ transition has not been recorded. We experimentally checked S/N on the peak position of the line and found it to be insufficient for detection. As it takes almost a full day to measure a line in fluorescence spectroscopy, we did not perform this measurement.

We have followed the referees suggestions and devised a new Figure 2 that explicitly transports this message: the panel for the $1/2 \rightarrow 3/2$ transition is added, indicating the measured datapoint on the line center, that coincides with the baseline. The frequency "ruler" in panel a now extends over the full width of panel b.

We have also followed the referee's suggestion and added the term "unresolved" below the label "O-center".

As the requested horizontal extension of the figure would lead to a further reduced size of the individual panels and datapoints, we suggest to "wrap" the level diagram into a new vertical line and have merged the content of Figure 2 with Table 1, maintaining the overall surface of graphical elements. We have adapted the main text and captions to accommodate this change in figure layout.

8. Fig. 4: we accept the authors request to keep this panel in the manuscript; the last sentence in the caption should be changed to "The (very narrow) quadrupole structure is not resolved." to be more precise.

Reply.
We followed the referee's suggestion and added "No substructure is resolved" to

the caption. We prefer to not use the term "very narrow", as the linewidth of the quadrupole structure remains speculative until it is resolved.

Referee #2 (Remarks to the Author):

The authors have provided comprehensive point-by-point responses to the reviewers' comments, supplemented and revised relevant content as required, and added explanatory notes. The revised manuscript features more rigorous presentation. The research findings reported in this paper represent a significant advance in the field of thorium-based solid-state nuclear clocks, and the revised manuscript is suitable for publication in this journal.

We thank the reviewer for the assessment and support.

Referee #3 (Remarks to the Author):

The authors have included most of my suggestions. Some of which they didn't presumably because my intentions were not clear. I am trying again below. Reading the manuscript again, I noticed two more issues that could be solved to improve it.

- The manuscript still misses a discussion of how to achieve the prospects given in the first sentence, that is the promise of an "optical nuclear clock of very high accuracy and stability". All parameters are far away from that and one would expect at least some speculations how the many orders of magnitude could be gained in terms of linewidth, systematics and stability. Such a discussion would be helpful even if it was speculative at this time. "... we do not expect to jump over the frequency instability of high-finesse optical cavities at short times." Not even the best optical clocks can do that. However, they operate with typically 1e6 atoms while the potential number of addressable Th-229 could at the mol scale. Stability scales as sqrt(N). To me this is enough motivation to speculate.

Reply:
We would like to point out that this discussion was already included in the revised version of the manuscript: In the section "Discussion and Outlook" starting with "In the future, this could enable the realization of a nuclear clock...". It explicitly indicates the scaling of the shot noise limited instability of an absorption based clock which scales with$\sigma(\tau) \approx \Delta\nu/(\nu_0 A(\dot{N}_\gamma \tau)^{1/2})$. The high number of nuclei enters through the absorption fraction A. In our estimates of realistic near-future improvements, this instability does not surpass high-finesse optical

cavities at short timescales.

We now start this discussion as a separate paragraph to make it more visible to the reader. We also now cite two very recent preprint manuscripts submitted after this work, that already implement a clock-like feedback cycle, using absorption spectroscopy and present a more detailed perspective on current and future performances.

- Fig1. is the MCP imaged on the CCD camera or just detected as the caption says? I do not recognize a lens for imaging.

Reply:
The camera has an objective and images the phosphorescence from the phosphor screen on the back of the MCP. We have added this information to the caption of figure 1.

- Line scan duration: "...crystal is periodically illuminated for 800 s, followed by a 800 s detection interval..."; "...scanned in 50 kHz steps over the ≈ 1 GHz rang..."; "Each transition is scanned twice...". Adding the numbers I conclude one line scan too 2yrs of uninterrupted operation. Later is says "The operation of an optical clock requires a signal to stabilize a laser to the reference frequency." Does that mean the servo bandwidth is limited to 1/2 years? Please clarify how long the line scans really take.

Reply:
The span of the quadrupole splitting (Table 1 and Fig. 2) is ≈ 500 MHz and since the laser frequency was scanned in both directions (blue-to-red and red-to-blue) the total span would be ≈ 1 GHz. With excitation and detection cycles used in fluorescence measurement that would require 2 years to cover the whole range.

For scanning an individual component 15 h are required (17 points for each line with 1600 s of excitation and detection for each data point in each scan direction). Therefore, the total time required in fluorescence measurement for all 5 lines is 75 h (5 × 15 h).

The referee is correct that fluorescence detection would strongly limit the servo bandwidth in clock operation, showing the importance of absorption spectroscopy as introduced in this work.

To avoid giving the impression that we have explored the full 1 GHz range in a continuous fluorescence scan, we have changed the wording to "the frequency is scanned in 50 kHz steps over the relevant frequency ranges of the quadrupole splitting in Th:CaF_2 reported by [3, 6]

- Again, shot noise:" The absorption and the error signals shown in Fig. 3 and Fig. 2c (absorption measurement of 6 lines) are not normalized." I think they are. This is what per cent = normalized to 100, literally means. "In this case

the lock-in technique is often used to reduce an influence of a background..." The lock-in is a low pass filter that reduces the bandwidth. It does not remove the shot noise. The latter is proportional to the bandwidth Bw. In order to verify the magnitude of the error bars in fig.2b/c I would need the number of photons during 1/Bw. Because the signals normalized, this important piece of information is lost. At least I can not find it in the caption.

The referee is correct that relative (i.e. normalized) absorption and normalized fluorescence (to the strength of the strongest line) is presented. The dominant noise contributions are different for the two spectroscopy methods. In the case of absorption, it is predominantly shot noise in the detected number of transmitted photons ($\approx 6 \cdot 10^6$ photons per second, given in the discussion). The influence of laser power fluctuations and drift is largely eliminated by the lock-in detection. For the fluorescence spectroscopy, the signal (≈ 50 photons per second for the strongest component) is detected on top of a constant radio luminescence and Cherenkov background of around 160 cps. Again, shot noise in the observed photon counts is dominant, with a contribution from the fluorescence photons and a bigger contribution from the luminescence background.

We have added these numbers in the sections on fluorescence measurements and absorption measurements.

- Again: "the optical transmission... at the nuclear transition wavelength was measured to be $\approx 40\%$". Technically correct but language wise very confusing if one does not mention that the absorption is also$\approx 40\%$ off resonance. Reading this I would falsely conclude that the absorption depth is $\approx 40\%$.This statement comes before it is clear that this dip is actually less than 1%.

Reply:
To avoid this confusion we replace 'the optical transmission ... at the nuclear transition' with 'the optical transmission ...in the range of several nanometers around the nuclear transition' in the text.

- Again, Cauchy: I have learned that the EFG shift is $1/r^3$ weighted with the probability r^2 this leads to a logarithmically diverging shift, just as with the Cauchy distribution. Therefore, the changes made to the manuscript do not solve resolve this issue, at least not for me, but even deepened it.

Reply:

In our understanding from the first round of reviews, the main concern of the referee stems from the undefined expectation value and variance of the Cauchy distribution. However, we have shown that this distribution can be derived with a physically well motivated reasoning. In a real crystal, this divergence

is regulated by physical constraints. An analogous divergence is discussed by Stoneham [Rev. Mod. Phys. 41, (1969), p.93] for inhomogeneous strain broadening, who notes that a distance cutoff is needed only to define the moments of such a distribution, not the lineshape used to describe the observed spectrum. Furthermore, the Cauchy distribution depends on two physically and statistically meaningful parameters, the location parameter and the half-width. In our case these are the two parameters (V_{zz} and δV_{zz}) we extract and report. We have added a sentence to the manuscript clarifying this point.

- Again, the suppression of the D-centers. I learned from the authors reply why the D-centers are not quadratically suppressed as everyone would expect. However, the information that it is due to clustering and that the lines are not normalized to different 100% levels is only given to me, not to the reader. At least I didn't find it in the caption.

Reply:
We have modified the caption of Fig. 2 to clarify that we applied a common normalization to all fluorescence signals. In this paper, we did not study the relative abundance of the two trapping sites and therefore do not speculate about a microscopic explanation. Such a study would require measurements with multiple different crystals, as it has been done previously in T. Hiraki, et al. arXiv:2509.00041.
